# Identification of new regulators through transcriptome analysis that regulate anthocyanin biosynthesis in apple leaves at low temperatures

**DOI:** 10.1371/journal.pone.0210672

**Published:** 2019-01-29

**Authors:** Tingting Song, Keting Li, Ting Wu, Yi Wang, Xinzhong Zhang, Xuefeng Xu, Yuncong Yao, Zhenhai Han

**Affiliations:** 1 College of Horticulture, China Agricultural University, Beijing, China; 2 Plant Science and Technology College, Beijing University of Agriculture, Beijing, China; 3 Beijing Collaborative Innovation Center for Eco-Environmental Improvement with Forestry and Fruit Trees, Beijing, China; Zhejiang University, CHINA

## Abstract

Anthocyanin pigments play many roles in plants, including providing protection against biotic and abiotic stresses. To identify new regulatory genes in apple (*Malus domestica*) that may be involved in regulating low temperature induced anthocyanin biosynthesis, we performed RNA-seq analysis of leaves from the ‘Gala’ apple cultivar following exposure to a low temperature (16 °C). A visible red color appeared on the upper leaves and the anthocyanin content increased significantly after the low temperature treatment. Genes from the flavonoid biosynthesis pathway were significantly enriched among the differentially expressed genes, and the expression of several transcription factors was shown by WGCNA (weighted gene co-expression network analysis) to correlate with anthocyanin accumulation, including members of the MYB, MADS, WRKY, WD40, Zinc Finger and HB-ZIP families. Three MYB transcription factors (*MdMYB12*, *MdMYB22* and *MdMYB114*), which had several CBF/DREB response elements in their promoters, were significantly induced by low temperature exposure and their expression also correlated highly with anthocyanin accumulation. We hypothesize that they may act as regulators of anthocyanin biosynthesis and be regulated by CBF/DREB transcription factors in apple leaves under low temperature conditions. The analyses presented here provide insights into the molecular mechanisms underlying anthocyanin accumulation during low temperature exposure.

## Introduction

Anthocyanins are pigments in the flavonoid family of phenylpropanoid compounds that are responsible for the blue, purple, and red colors of leaves, flowers and fruits [1]. Flavonoids play diverse roles in plants, including providing protection against UV light and pathogens, and attracting animal pollinators [2,3]. As dietary components they also have beneficial effects on human health as they provide a source of antioxidants, reduce the incidence of coronary heart disease and exhibit anticancer activity [2].

The anthocyanin biosynthesis pathway and associated genes have been studied in numerous plant species, such as *Arabidopsis thaliana* and tobacco (*Nicotiana benthamiana*) [4,5], as well as many fruit trees, such as apple (*Malus domestica*) [6,7], grape (*Vitis vinifera*) [8], pear (*Pyrus communis* L.) [9] and tea (*Camellia sinensis L*.) [10]. The most characterized biosynthetic genes include phenylalanine ammonia lyase (*PAL*), chalcone synthase (*CHS*), chalcone isomerase (*CHI*), flavanone 3-hydroxylase (*F3H*), flavonoid 3’-hydroxylase (*F3*’*H*), dihydroflavonol 4-reductase (*DFR*), anthocyanin synthase (*ANS*) and UDP-glucose: flavonoid 3-*O*-glucosyltransferase (*UFGT*) [4,5]. Many studies have shown that the expression of anthocyanin biosynthesis genes is spatially and temporally controlled by transcription factors, and particularly by a conserved MYB-bHLH-WD40/WDR (MBW) complex [11,12]. This complex binds to the promoters of anthocyanin biosynthetic genes and induces their expression during development, or in response to a range of environmental conditions [3,11,13]. In apple (*Malus domestica*), the anthocyanin biosynthetic pathway has been shown to be controlled by the MYB transcription factors, *MdMYB1*, *MdMYB10*, and *MdMYBA* [6,14,15]. Studies with transgenic plants have also shown that these genes are key regulators of anthocyanin accumulation and fruit coloration through their interaction with MdbHLH3 and WD40 proteins, and binding to the promoters of late anthocyanin biosynthetic genes, such as *MdANS* and *MdUFGT* [6,14,15,16].

Environmental factors, such as drought, high salt levels, and high or low temperatures, represent major abiotic stresses for plants [15]. For example, low temperatures affect growth and development and limit geographical distribution and crop yield [17,18]. Low temperatures are known to induce anthocyanin biosynthesis, and both low temperature stress and anthocyanin levels in leaves have been reported to correlate with low temperature tolerance in some *A*. *thaliana* ecotypes [19]. In addition, anthocyanins in combination with flavonols help limit the over-excitation of chlorophyll under low temperature conditions [18,20,21]. Taken together, these results support a role for anthocyanins in the defense against low temperature-induced damage.

It has been well documented that low temperatures stimulate anthocyanin accumulation by up-regulating the expression of anthocyanin biosynthetic genes [22–26]. Furthermore, it is known that the MBW complex, especially the bHLH and MYB components, is involved in modulating anthocyanin accumulation at low temperatures. *MdMYBA*, which regulates anthocyanin biosynthesis in apple skin, binds specifically to the ANS promoter and activates anthocyanin accumulation under low temperature conditions [14], while MdbHLH3 binds to the promoters of the anthocyanin biosynthetic genes, *MdDFR* and *MdUFGT*, and the regulatory *MdMYB1* gene to activate their expression [27]. A MYB transcription factor, BoPAP1, has been proposed to activate anthocyanin accumulation by enhancing the expression of the *C4H*, *F3H*, *DFR*, *ANS* and *UFGT* in the purple kale (*Brassica oleracea*) during low temperatures [28]. Recently, a MYB transcription factor MdMYB15L were found that it functioned as a repressor and negatively regulated anthocyanin by interacting with MdbHLH33 in the cold signaling [29]. Other regulatory genes that have been found to regulate low temperature-induced anthocyanin accumulation in *A*. *thaliana* seedlings include HY5 and HYH [30]. In addition, a NAC transcription factor (TF) is selectively induced by cold in blood oranges (*Citrus × sinensis*) but not in common oranges [31,32], and a SUMO E3 ligase, MdSIZ1, was reported to directly sumoylate MdMYB1 proteins under moderately low temperature (17 °C) in apple [33].

Previous study showed that 16 °C low temperature and 30 °C high temperature promoted or inhibited anthocyanin accumulation in apple leaves, respectively [34]. In the current study, we used RNA-seq analysis to study variation in gene expression in the apple cultivar ‘Royal Gala’ during moderate 16 °C low temperature treatment, in order to identify candidate regulatory genes associated with low temperature induced anthocyanin biosynthesis. We describe the use of an unbiased network analysis tool to elucidate modules of co-expressed genes that are rapidly and abundantly expressed after low temperature treatment, and that are also associated with anthocyanin accumulation. We propose that MYB TFs play an important role in the synergistic regulation of low temperature responses and anthocyanin accumulation.

## Materials and methods

### Plant materials

Stem explants of *Malus domestica* cv. ‘Royal Gala’ were excised from one-year-old branches before spring bud germination, and cultured on Murashige and Skoog medium [35] supplemented with 0.4 mg/L 6-benzylaminopurine (6-BA) and 0.05 mg/L 1-naphthylacetic acid (NAA) at 23 °C with a 16 h light (200 μmol. s^−1.^ m^–2^) /8 h dark period for 30 d to induce leaf reproduction before collection. Temperature treatments involved exposing whole plants to 16 °C and then sampling 30-day-old leaves at 0 h, 6 h, 1 d, 3 d and 5 d following treatment, and after which leaves were frozen in liquid nitrogen and stored at -80 °C prior to anthocyanin or RNA extraction.

### RNA quantification and quality analysis

RNA integrity and purity was assessed using 1% agarose gels, a Nano Photometer spectrophotometer (IMPLEN, CA, USA), and a RNA Nano 6000 Assay Kit with the Bioanalyzer 2100 system (Agilent Technologies, CA, USA). RNA concentration was measured using a Qubit RNA Assay Kit in a Qubit 2.0 Fluorometer (Life Technologies, CA, USA).

### Library preparation for sequencing

A total of 3 μg RNA per sample was used to generate RNA-Seq libraries using the NEBNextUltra RNA Library Prep Kit for Illumina (NEB, USA) following the manufacturer’s recommendations. Index codes were added to label each sample. In order to preferentially select cDNA fragments 150–200 bp in length, the library fragments were purified with an AMPure XP system (Beckman Coulter, Beverly, USA), then 3 μL USER Enzyme (NEB, USA) was used with size-selected, adaptor-ligated cDNA at 37 °C for 15 min, followed by 5 min at 95 °C. PCR was performed with the Phusion High-Fidelity DNA polymerase, Universal PCR primers and Index (X) Primers. Finally, PCR products were purified (AMPure XP system) and library quality was assessed using an Agilent Bioanalyzer 2100 system. The library preparations were sequenced on an Illumina Hiseq platform (Illumina, USA), and paired-end reads were generated.

### Read mapping to the reference genome and quantification of gene expression

An index of the reference genome was built using Bowtie v2.2.3 [36] and paired-end clean reads were aligned to the apple reference genome [37] using TopHat v2.0.12 [38]. HTSeq v0.6.1 (https://pypi.python.org/pypi/HTSeq) was used to count the read numbers mapped to each gene [39]. The FPKM (Fragments Per Kilobase of transcript per Million mapped reads) method was used to investigate differential gene expression at different leaf developmental stages, and the FPKM of each gene was calculated based on the length of the gene and read counts mapped to the gene.

### Differential expression analysis

Differential expression analysis of four groups (four biological replicates per group) was performed using the DESeq R package (1.18.0) (http://www.bioconductor.org/packages/release/bioc/html/DESeq.html) [40]. DESeq provides statistical routines for determining differences in digital gene expression data using a model based on the negative binomial distribution. The resulting *P*-values were adjusted using the Benjamini and Hochberg approach [41] for controlling the false discovery rate. Genes with an adjusted *P*-value <0.05 were considered to be differentially expressed [42].

### Gene ontology (GO) and Kyoto Encyclopedia of Genes and Genomes (KEGG) enrichment analysis of differentially expressed genes (DEGs)

The Blast2GO software package was used to identify GO enriched terms [43]. GO terms with a corrected *P*< 0.05 were considered significantly enriched with DEGs [43]. KOBAS software was used to test the statistical enrichment of DEGs among KEGG pathways (http://www.genome.jp/kegg/) [44].

#### Identification of co-expression modules

The R package WGCNA (weighted gene co-expression network analysis) [45,46] was used to identify modules of highly correlated genes based on the FPKM data. The DCGL R package [47] was used to filter the genes based on gene expression and variations. The pickSoft Threshold function in the WGCNA package was used with a soft thresholding power of 7, which was interpreted as a soft threshold for the correlation matrix. The resulting adjacency matrix was then converted to a topological overlap (TO) matrix using the TOMsimilarity algorithm [48]. Genes were hierarchically clustered based on TOMsimilarity. The Dynamic Hybrid Tree Cut algorithm was then used to cut the hierarchal clustering tree and defined modules as branches from the tree cutting [48]. We summarized the expression profile of each module by representing it as the first principal component (referred to as a module eigengene). Modules whose eigengenes were highly correlated (correlation >0.8) were merged.

#### High Performance Liquid Chromatography (HPLC) analysis

Frozen apple leaf samples (approximately 0.8–1.0 g fresh weight) were ground in 10 mL extraction solution (methanol: water: formic acid: trifluoroacetic acid = 70: 27: 2: 1) and incubated at 4 °C in the dark for 72 h, with shaking every 6 h. The supernatant was passed through filter paper and then through a 0.22 μm Millipore filter (Billerica, MA, USA). For the HPLC analysis, trifluoroacetic acid: formic acid: water (0.1: 2: 97.9) was used as mobile phase A and trifluoroacetic acid: formic acid: acetonitrile: water (0.1: 2: 48: 49.9) was used as mobile phase B. The gradients used were as follows: 0 min, 30% B; 10 min, 40% B; 50 min, 55% B; 70 min, 60% B; 30 min, 80% B. Absorbance was measured at 520 nm for anthocyanins and 280 nm for proanthocyanidins (PAs) [49]. All samples were analyzed in three biological triplicates (extracted from three different batches of leaves).

### RNA extraction and quantitative real time (qRT)-PCR analysis

Total RNA was extracted from apple leaves using an RNA Extraction Kit (Aidlab, Beijing, China) according to the manufacturer’s instructions. DNase I (TaKaRa, Ohtsu, Japan) was added to remove genomic DNA, and the samples were converted to cDNA using the Access RT-PCR System (Promega, USA), according to the manufacturer’s instructions. Gene expression levels were analyzed using qRT-PCR and the SYBR Green qPCR Mix (TaKaRa, Ohtsu, Japan) with a Bio-Rad CFX96 Real-Time PCR System (BIO-RAD, USA), according to the manufacturers’ instructions. The PCR primers (S1 Table) were designed using NCBI Primer BLAST (https://www.ncbi.nlm.nih.gov/tools/primer-blast/). qRT-PCR analysis was carried out in a total volume of 20 μL containing 9 μL of 2×SYBR Green qPCR Mix (TaKaRa, Ohtsu, Japan), 0.1 μM specific primers (each), and 100 ng of template cDNA. The reaction mixtures were heated to 95 °C for 30 s, followed by 39 cycles at 95 °C for 10 s, 50–59 °C for 15 s, and 72 °C for 30 s. A melting curve was generated for each sample at the end of each run to ensure the purity of the amplified products. The transcript levels were normalized using the *Malus domestica 18S ribosomal RNA* gene (GenBank ID DQ341382) as the internal control and calculated using the 2^^(−ΔΔCt)^ analysis method [50].

### Phylogenetic and promoter analysis

Phylogenetic and molecular evolutionary analyses were conducted using MEGA version 5.1, with a minimum evolution phylogeny test with 1,000 bootstrap replicates [51]. *Cis*-element analysis of MYB TF promoters was performed using the PLACE database (http://www.dna.affrc.go.jp/PLACE/signalscan.html).

### Data analysis

All data were analyzed using one-way ANOVA followed by Tukey’s multiple range test to compare differences among the experimental sites at *P*<0.05. OriginPro8, Microsoft Excel 2007, Data Processing System (DPS) software 7.05 and IBM SPSS Statistics 22 were used for analysis.

## Results

### Effect of low temperature on apple leaf coloration

**Fig 1:**
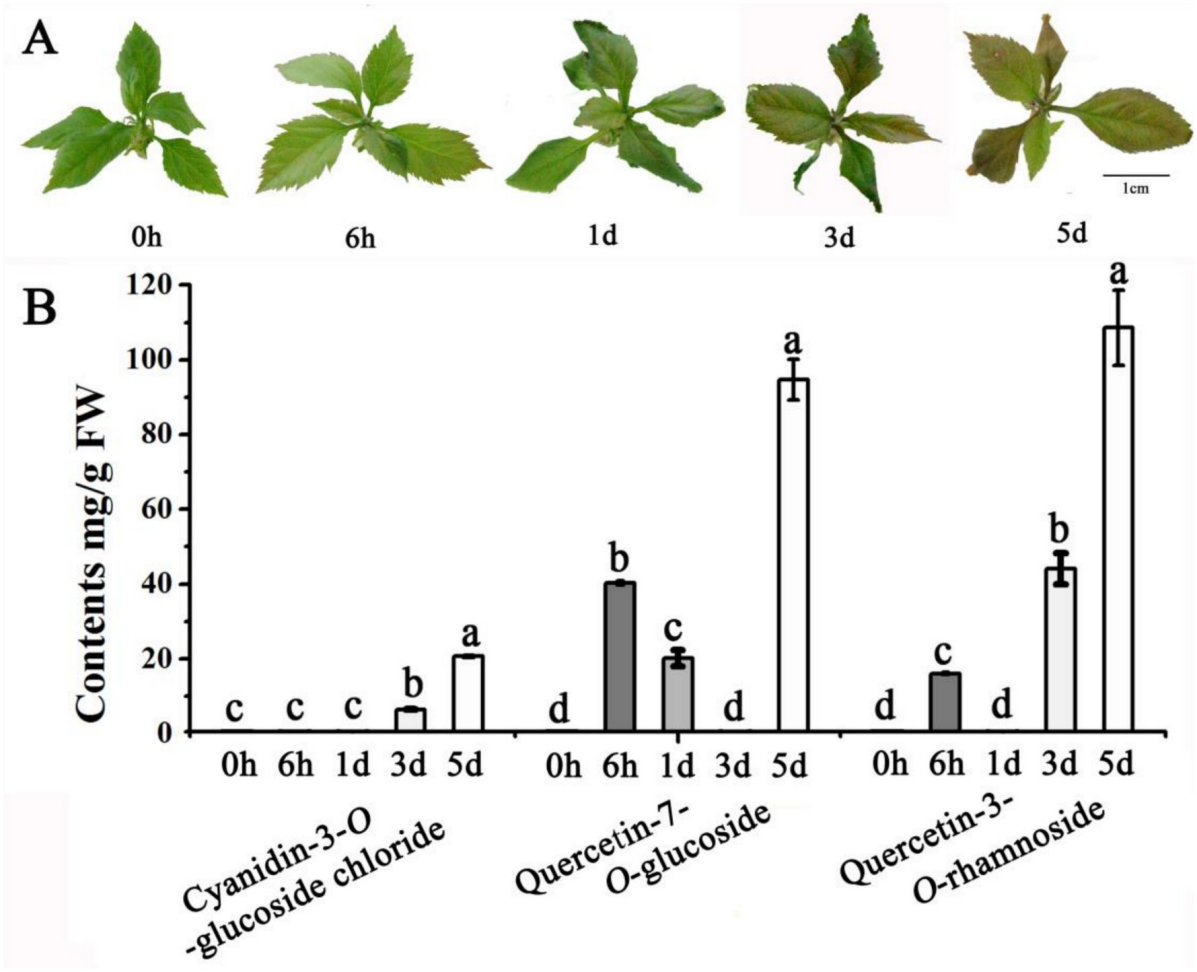
Effects of low temperature treatment on accumulation of anthocyanin and flavonols in apple leaves (*Malus domestica* ‘Gala’). (A) The phenotype of ‘Gala’ apple foliage after low temperature treatment. Scale bars = 1cm. (B) Flavonoid profiles in apple leaves during low temperature treatment. Different letters above the bars indicate significantly different values (*P* < 0.05) calculated using one-way analysis of variance (ANOVA) followed by a Tukey’s multiple range test.

To understand the effect of low temperature on apple leaf coloration, we placed 30-day-old apple plantlets at 16 °C for 0 h, 6 h, 1 d, 3 d and 5 d. As shown in Fig 1A, a visible red color appeared on the margin of the upper leaves after 1d of 16 °C exposure, and this color became strong after 5 d exposure. HPLC analysis revealed that anthocyanin levels (cyanidin-3-*O*-glucoside chloride) increased from 0.0 to 20.70 μg/g after the 5 d low temperature treatment. Besides, two flavonols, quercetin-7-*O*-glucoside and quercetin-3-*O*-rhamnoside (avicularin), increased significantly as well (Fig 1B).

### RNA-seq analysis of apple leaves under low temperature treatment

We performed RNA-seq analysis of the low temperature treated apple leaves to identify those involved in regulating anthocyanin biosynthesis. The number of raw reads for each library ranged from 21 to 34 million and when the clean reads were mapped to the assembled ‘Golden Delicious’ reference apple genome, the mapping rate varied from 88.83% to 90.56% (S2 Table) [37]. A Pearson correlation analysis indicated that all libraries from the biological replicates of each low temperature treatment showed highly consistent transcriptome profiles (r^2^ = 0.8456–0.9794; see S1 Fig). Mapping region classifications included exons, introns, intergenic regions, and spliced regions. Among all the libraries, the proportion of exons was the highest, ranging from 90.15% to 93.08%, and the proportion of introns was the lowest, ranging from 2.93% to 4.80% (S2 Fig). In addition, the Q20 and Q30 percentages (a Q-score of 20 and 30 corresponds to an error rate of 1 per 1000) of all libraries were >92% (S2 Table), which indicated that the RNA and sequence quality were high, and that the data provided a reliable basis for further studies of gene expression.

### Changes in gene expression profiles during low temperature treatment

**Fig 2:**
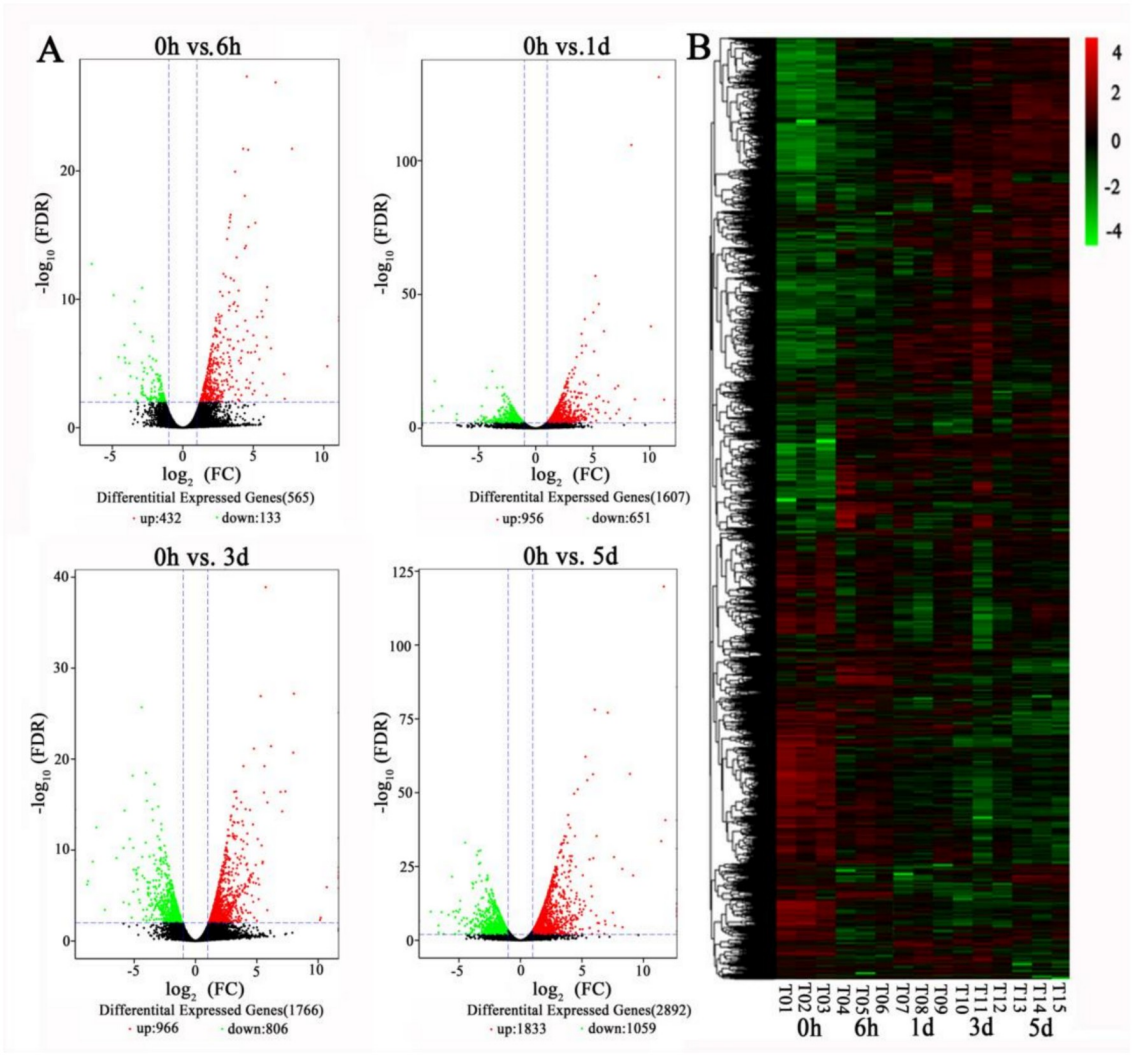
RNA-seq analysis of leaves from the ‘Gala’ apple cultivar during low temperature treatment. (A) Volcano plot of the RNAS-Seq data showing the differentially expressed genes (DEGs) in red and green. The X-axis represents the fold change in 0 h versus 6 h, 0 h versus 1 d, 0 h versus 3 d and 0 h versus 5 d (on a log_2_ scale) samples, and the Y-axis represents the negative -log_10_ transformed *P*-values (*P*< 0.05) of the t-test for differences between the samples. (B) Cluster analysis of DEGs during low temperature treatment.

To evaluate differential gene expression during the low temperature treatment, FPKM values were used to investigate differences in transcript abundance. Stringent values of ratio >2.0, *P* value <0.05 and ratio <0.5, *P* value <0.05 were used as the threshold to assess significant differences in gene expression. A total of 21192 differentially expressed genes (DEGs), including 12296 up-regulated and 8896 down-regulated were identified during low temperature treatment in apple leaves. We found that 432 genes were up-regulated and 133 genes were down-regulated at 0 h versus 6 h; 956 genes were up-regulated and 651 genes were down-regulated at 0 h versus 1 d; 966 genes were up-regulated and 806 genes were down-regulated at 0 h versus 3 d; and 1,833 genes were up-regulated and 1,059 were down-regulated at 0 h versus 5 d (Fig 2A). We noted that the comparison with largest number of differentially expressed genes (0 h versus 5 d) corresponded the peak of anthocyanin abundance at 5 d (Fig 2B).

### GO annotation and KEGG pathway analyses

**Fig 3:**
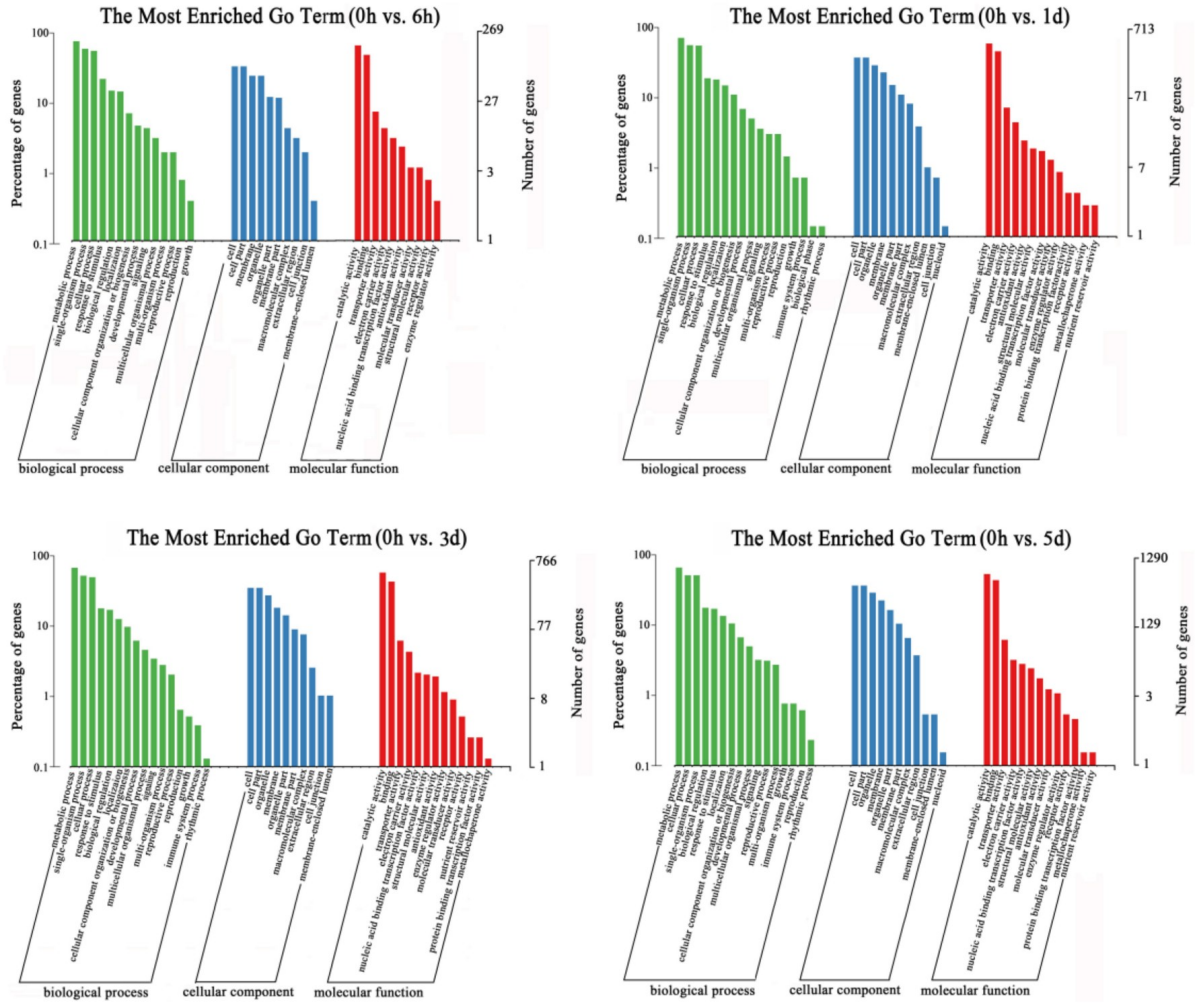
Histogram of gene ontology (GO) classifications of genes expressed in ‘Gala’ leaves during low temperature treatment. The unigenes were placed in three main categories: ‘biological process’, ‘cellular component’ and ‘molecular function’. The left and right-hand y-axes indicate the percentage and number of annotated unigenes, respectively.

**Fig 4:**
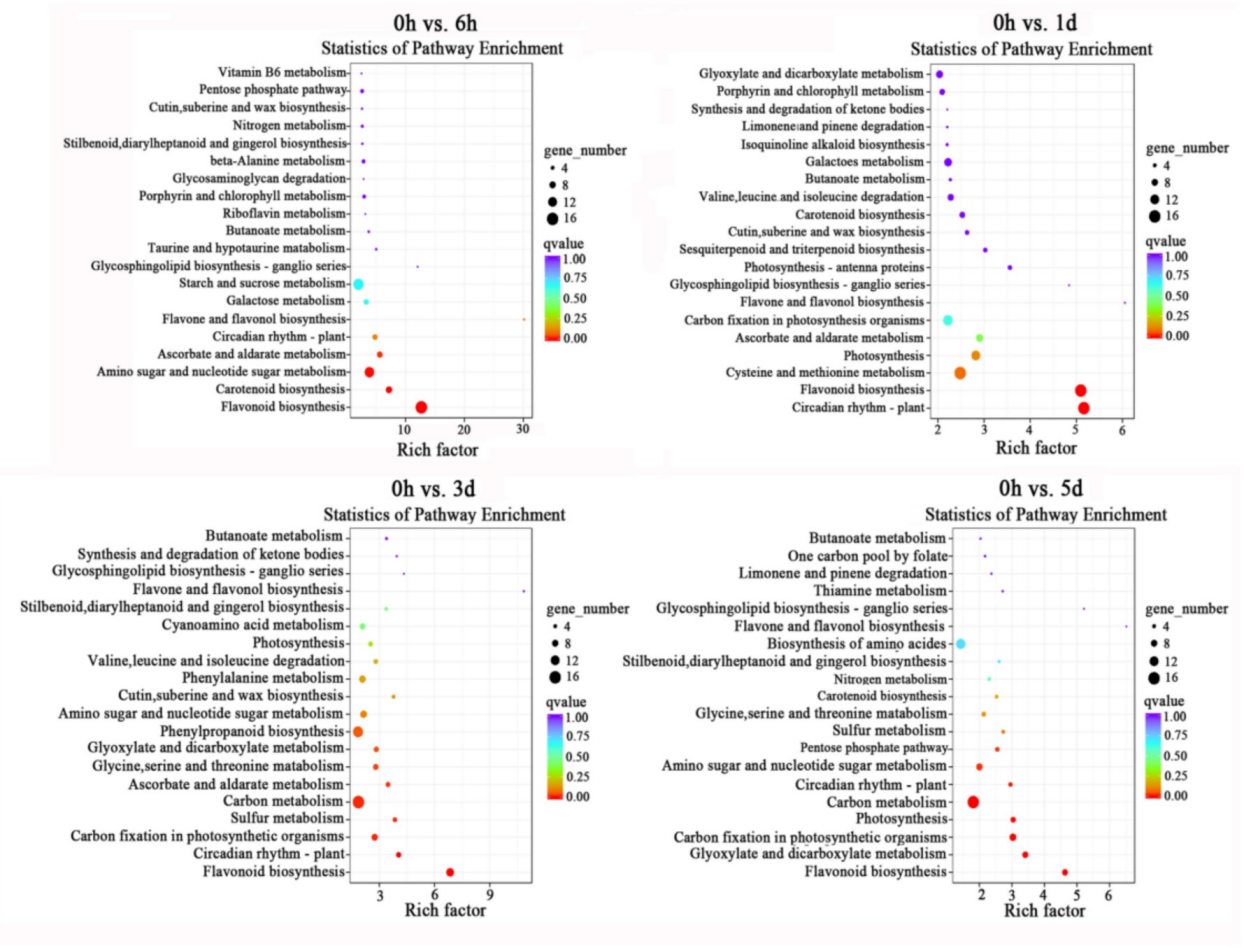
KEGG (Kyoto Encyclopedia of Genes and Genomes) pathway enrichment of differentially expressed genes (DEGs) in 0 h versus 6h, 0 h versus 1 d, 0 h versus 3 d and 0 h versus 5 d samples.

GO-based term classification was performed to provide insights into DEG function. The numbers of DEGs involved in ‘metabolic process’, ‘cellular process’, ‘cell part’, ‘cell’, ‘catalytic activity’, and ‘binding’ were significantly higher during the low temperature treatment (Fig 3). When KEGG pathway and enrichment analyses were used to classify the DEGs and highlight biological associations, the flavonoid biosynthesis, amino sugar and nucleotide sugar metabolic pathways were significantly enriched in DEGs at 0 h versus 6 h. Furthermore, the flavonoid biosynthesis pathway contained the largest number of DEGs at 0 h versus 1 d, and the flavonoid biosynthesis and carbon metabolism pathways were both enriched in DEGs at 0 h versus 1 d and 0 h versus 5 d (Fig 4). The genes from the flavonoid biosynthesis pathway that were enriched by the low temperature treatment are listed in S3 Table. The results were consistent with flavonoid biosynthesis being involved in low temperature stress tolerance and provided a basis for identifying candidate genes related to anthocyanin biosynthesis.

### Identification of WGCNA modules associated with anthocyanin biosynthesis

**Fig 5:**
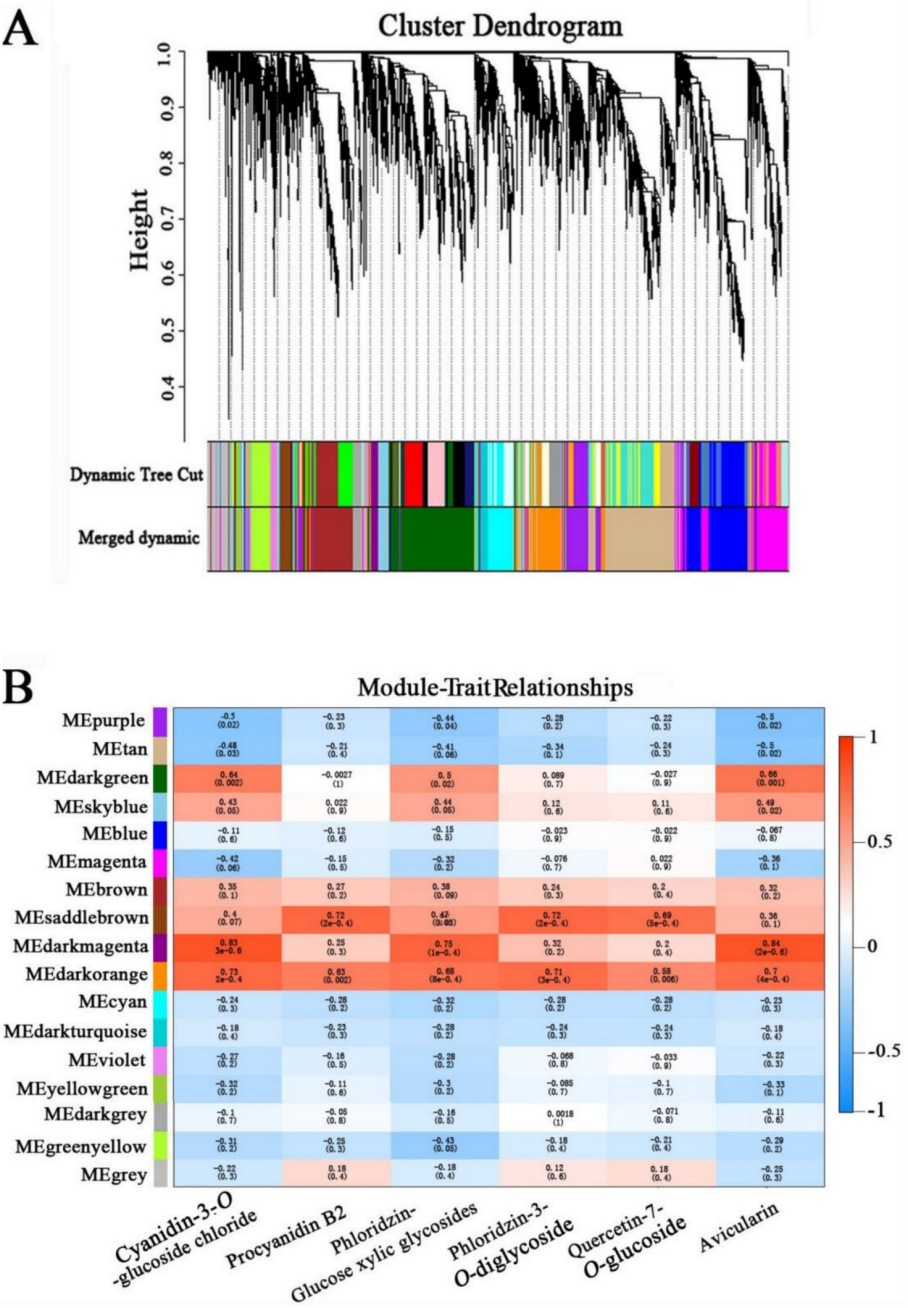
Network analysis dendrogram showing modules identified by WGCNA (weighted gene co-expression network analysis). (A) Dendrogram with color annotation. (B) Module-anthocyanin weight correlations and corresponding *P*-values (in parentheses). The left panel shows seven modules. The color scale on the right shows module-trait correlation from -1 (blue) to 1 (red).

WGCNA can be used to identify networks of functionally associated genes [52]. To identify the genes associated with anthocyanin biosynthesis during the low temperature treatment, a WGCNA was performed with all 3,296 DEGs, resulting in 17 WGCNA modules (Fig 5). The largest module (‘Tan’) contained 543 genes, while the smallest module (‘Violet’) contained 44 genes. Analysis of the module-trait relationships revealed that the ‘Darkmagenta’ (r = 0.83, *p* = 3e- 06) and ‘Darkorange’ (r = 0.73, *p* = 2e- 04) modules were highly correlated with anthocyanin accumulation in all modules (Fig 5A and 5B). A total of 17 TFs were annotated in the ‘Darkmagenta’ and ‘Darkorange’ modules. As shown in S4 and S5 Tables, several TFs highly positively correlated with anthocyanin accumulation were found amongst these 17 candidate anthocyanin regulatory genes, including 11 MYB TFs (myeloblastosis-related), 2 MADS TFs (MCM1 from the *Saccharomyces cerevisiae*, AGAMOUS from the *Arabidopsis thaliana*, DEFICIENS from the *Antirrhinum majus*, SRF from the *Homo sapiens*), 1 WRKY TF (WRKYGQK sequence), 1 WD40 (tryptophan-aspartic acid (W-D) 40 repeat), 1 Zinc Finger and 1 HD-ZIP TF (homeodomain leucine zipper). We hypothesized that these putative regulatory genes may participate in pigment accumulation in response to low temperatures.

### Genes related to flavonoid biosynthesis

**Fig 6:**
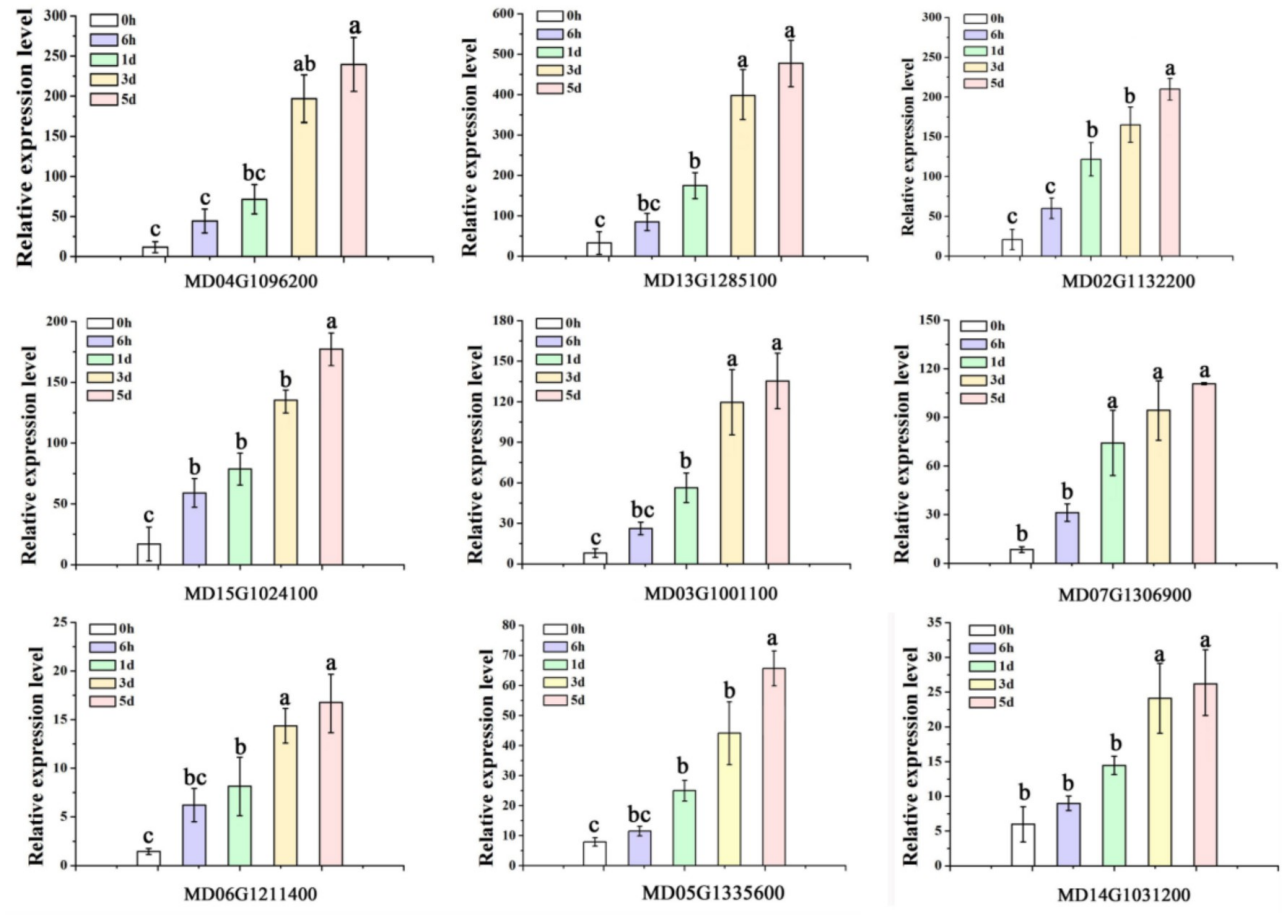
Verification of the expression of 12 differentially expressed genes (DEGs) by qRT-RCR. The expression levels of flavonoid regulatory and biosynthetic genes were calculated using CFX-Manager-3-1 following the manufacturer’s instructions (Bio-Rad). Different letters above the bars indicate significantly different values (*P* < 0.05) calculated using one-way analysis of variance (ANOVA) followed by a Tukey’s multiple range test.

Notably, all the genes known to be involved in the flavonoid biosynthetic pathway were clustered in the ‘Darkmagenta’ and ‘Darkorange’ modules in the WGCNA analysis. Data from qRT-PCR analysis of the expression of eight anthocyanin biosynthetic genes, *MdPAL1* (MD04G1096200), *MdCHS* (MD13G1285100), *MdF3H* (MD02G1132200), *MdDFR* (MD15G1024100), *MdANS* (MD03G1001100), *MdUFGT* (MD07G1306900), *MdLAR1* (MD06G1211400), *MdANR1* (MD05G1335600) were consistent with the data from RNA-seq analysis for the various low temperature treatment points (Fig 6). The expression patterns of the genes involved in flavonoid biosynthesis were quite similar to anthocyanin accumulation trend. The correlation analysis of qRT-PCR indicated these eight genes to be related to anthocyanin accumulation, with r^2^ values ranging from 0.737 (MD02G1132200) to 0.942 (MD04G1096200) at the *P*<0.05 significance level (Table 1).

**Table 1:** Correlation analysis between relative expression levels of genes and the accumulation of anthocyanins in apple leaves.

	**MD04G1096200**	**MD13G1285100**	**MD02G1132200**	**MD15G1024100**	**MD03G1001100**	**MD07G1306900**
	**PAL1**	**CHS**	**F3H**	**DFR**	**ANS**	**UFGT**
**Anthocyanin**	0.942**	**0.774**	**0.737**	**0.805**	**0.753**	**0.769**
	**MD06G1211400**	**MD05G1335600**	**MD14G1031200**	**MD03G1297100**	**MD15G1215500**	**MD17G1261000**
	**LAR**	**ANR**	**LWD2**	**Myb-related protein**	**MYB12**	**MYB113**
**Anthocyanin**	**0.835**	**0.838**	**0.747**	**0.878**	**0.821**	0.964**

** indicates extremely significant in Pearson’s way(*p* = 0.05)

### Candidate MYB TFs involved in regulating flavonoid biosynthesis

**Fig 7:**
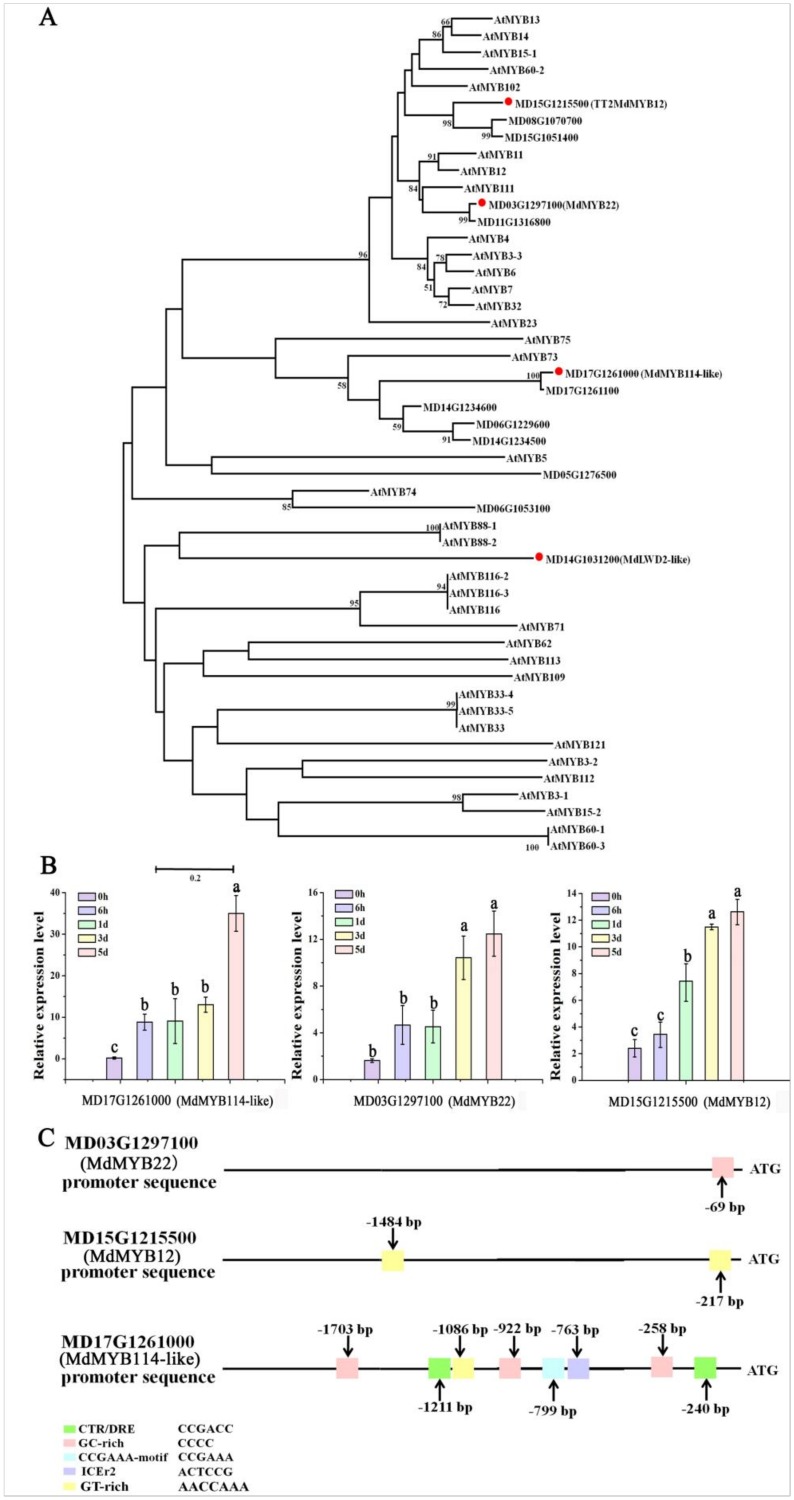
Analysis the candidate MYB transcription factors. (A) Phylogenetic analysis of MYB transcription factors in apple and their homologues in *Arabidopsis*. (B) The expression levels of MYBs genes were calculated using CFX-Manager-3-1 following the manufacturer’s instructions (Bio-Rad). Different letters above the bars indicate significantly different values (*P* < 0.05) calculated using one-way analysis of variance (ANOVA) followed by a Tukey’s multiple range test. (C) Schematic overview of low temperature response binding sites in MYBs promoters.

MYB TFs have been shown to play an important role in flavonoid biosynthesis [13] and were of particular interest in this study. We constructed a phylogenetic tree using *A*. *thaliana* MYB TF family members and putative apple MYB TFs identified in the ‘Darkmagenta’ and ‘Darkorange’ modules. MdMYB114-like (MD17G1261000) and MdMYB12 (MD15G1215500) showed a close relationship to AtMYB73 and AtMYB102, respectively, which have been reported to be involved in responses to salt and drought stress [53,54]. Furthermore, MdMYB22 (MD03G1297100) was closely related to AtMYB12, AtMYB111 and AtMYB11, which are all involved in flavonoid synthesis [55] (Fig 7A). The expression levels of *MdMYB22*, *MdMYB12* and *MdMYB114-like* were higher than the other MYB TFs and also showed a high correlation with anthocyanin abundance (MD15G1215500, 0.821; MD17G1261000, 0.964) (Fig 7B). These three TFs were therefore identified as strong candidates for regulating anthocyanin biosynthesis during low temperature stress. Finally, a *cis*-element analysis revealed several CBF/DREB response elements in the promoters of *MdMYB22*, *MdMYB12* and *MdMYB114-like*, suggesting that these genes are regulated by CBF/DREB TFs (Fig 7C).

## Discussion

Low temperatures significantly reduce the yield and productivity of crops [56] and the overall fitness of plants, and many species have evolved adaptive mechanisms to combat this stress [57]. As an example, one of the roles of anthocyanin pigments is to provide protection against biotic and abiotic stresses, and studies have shown their association with low temperature responses [2]. However, many details of the underlying regulatory networks remain to be elucidated. Here, we performed a transcriptome analysis of leaves from apple seedlings grown at low temperatures to identify TFs involved in the regulation of anthocyanin biosynthesis.

Recent studies have shown a correlation between flavonoid content and plant cold or low temperature tolerance, likely due to high antioxidant activity when scavenging reactive oxygen species (ROS) produced during cold or low temperature stress. It has been proposed that flavonoids can directly protect membranes and/or proteins from low temperature damage as they stabilize proteins *in vitro* by preventing their aggregation [58]. In our study, we demonstrated that anthocyanin accumulation in apple leaves is greatly increased by low temperatures, resulting in red color development after 5d of treatment. This suggests that the accumulation of anthocyanins may be important for low temperature tolerance in apple leaves. Flavonols in particular are known to play a role in protection against UV radiation and other environmental stresses [59], and indeed we observed that the flavonol content of ‘Gala’ leaves increased following the low temperature treatment.

It has been reported that in *A*. *thaliana* leaves, *PAL* and *CHS* transcript abundance increases in a light dependent manner when exposed to low temperature [60]. In addition, anthocyanins accumulate in vesicles and the transcript levels of *PAL*, *CHS*, *DFR*, and *UFGT* are strongly induced when red orange [*Citrus sinensis* (L.) Osbeck] is stored at 4 °C [32]. Furthermore, the expression of *CHS*, *ANS*, and *UFGT* was reported to be enhanced along with the accumulation of anthocyanins in the skin of apple fruit following low temperature treatment [23]. In crabapple (*Malus* crabapple) leaves, early anthocyanin biosynthetic genes (*McCHS*, *McF3H* and *McDFR*) are involved in the low-temperature induced anthocyanin accumulation [34,61]. Here, KEGG analysis indicated that the flavonoid biosynthetic pathway is upregulated in apple leaves following low temperature treatment, and the WGCNA analysis distributed almost all genes from the anthocyanin biosynthetic pathway into two modules: ‘Darkorange’ and ‘Darkmagenta’. Furthermore, qRT-PCR confirmed that *MdPAL*, *MdCHS*, *MdF3H*, *MdDFR*, *MdANS* and *MdUFGT* were strongly induced by low temperature treatment. We hypothesize that lower temperatures can trigger red coloration in the foliage by activating genes from the different stages of anthocyanin biosynthesis.

Anthocyanin biosynthesis is regulated at the transcriptional level by a set of TFs, including R2R3-MYB, bHLH and WD40, as well as members of several other TF families [6]. In order to identify regulatory genes involved in controlling anthocyanin biosynthesis in leaves during low temperatures, we classified the DEGs into 17 modules using WGCNA analysis, and identified two expression modules, ‘Darkorange’ and ‘Darkmagenta’, with a close relationship to anthocyanin accumulation. Interestingly, different kinds of TFs were present in ‘Darkorange’ and ‘Darkmagenta’ modules, including MADS, WRKY, Zinc-Finger and HD-ZIP members, examples of which have previously been implicated in the regulating low temperature response. In *Brassica rapa*, 19 BrMADS were found to show variable transcript abundance from a low temperature-treated whole-genome microarray data set. CsWRKY46 from cucumber conferred cold tolerance to transgenic plants and positively regulated the cold signaling pathway in an ABA-dependent manner. Overexpressing OsCTZFP8, a C2H2 zinc finger protein, exhibited cold tolerant phenotypes with significantly higher pollen fertilities and seed setting rates than non-transgenic control. The HD-Zip I homologous transcription factors HaHB1 from sunflower and AtHB13 from *Arabidopsis* were identified as playing a key role in cold response via the induction of proteins able to stabilize cell membranes [62–65]. These studies suggest that the MADS, WRKY, Zinc-Finger and HD-ZIP TFs identified in the ‘Darkorange’ and ‘Darkmagenta’ modules may be involved in low temperature induced anthocyanin biosynthesis in apple leaves, likely working together with a MBW complex.

Meanwhile, 11 out of 17 MYB TFs were present in these two modules. MYB proteins constitute a large family in plants and are characterized by the presence of a structurally conserved DNA binding domain, termed the MYB domain [66–68]. The R2R3-MYB subfamily is involved in a variety of biological functions, such as developmental regulation and responses to hormones and environmental factors [69–73], such as temperature. For example, *A*. *thaliana* plants overexpressing GmMYB76 or GmMYB177 transcription factors from soybean (*Glycine max*) showed improved salt and freezing tolerance compared with wild-type plants [74]. In rice (*Oryza sativa*), functional analysis revealed that overexpression of OsMYB30 transcription factors resulted in increased cold sensitivity, while an *osmyb30* knockout mutant showed increased cold tolerance [75].

MdMYB22, which was identified in our transcriptome analysis, has a high amino acid sequence identity to AtMYB12, AtMYB111 and AtMYB11 from *A*. *thaliana* [55]. These three *A*. *thaliana* proteins have been shown to be involved in flavonoid biosynthesis in both *A*. *thaliana* and tobacco, and we propose that MdMYB22 regulate anthocyanin biosynthesis in apple leaves. MdMYB114 and MdMYB12 were clustered with AtMYB73 and AtMYB102, respectively. Meanwhile, AtMYB73 and AtMYB102 have been proved that they are involved in salt and drought resistance in *A*. *thaliana*. So we deduced that MdMYB114 and MdMYB12 may have same function in stress resistance [53,54]. The expression results showing higher MdMYB12, MdMYB22 and MdMYB114 expression under low temperature conditions are consistent with these TFs acting as anthocyanin biosynthesis regulators. Interestingly, *cis*-element analysis showed that several CBF/DREB response elements were present in their promoters. Given that the function of MYB transcription factors in cold and low temperature stress responses has been suggested to be depended on the CBF/DREB pathway [76,77], we hypothesize that these MYB transcription factors are located downstream from CBF/DREB and promote anthocyanin accumulation by binding to the promoters of anthocyanin biosynthetic genes during low temperature conditions.

## Conclusions

In summary, exposure of apple leaves to low temperature treatment resulted in the accumulation of anthocyanins, suggesting a role for these pigments in low temperature tolerance. Transcriptome profiling of the low temperature treated leaves and subsequent WGCNA revealed two gene expression modules significantly associated with anthocyanin accumulation. These modules included three MYB TF genes that may play a role in low temperature-induced anthocyanin biosynthesis and be located downstream from CBF/DREB TFs. The analyses presented here provide insights into the molecular mechanisms underlying anthocyanin accumulation during low temperature conditions. As a future target, we will focus on the function of the MYB and CBF/DREB TFs, together with other regulators identified in the WGCNA networks.

## Supporting information

S1 Fig Pearson correlation analysis. Heat map of the correlations between biological replicates. The PCC (Pearson correlation coefficient) values are quantitative indicators of relative expression levels of all genes in each sample.
(DOC)

S2 Fig The distribution of cleaned reads mapped to the reference genome. The terms exon, intron and intergenic refer to the percentage of cleaned reads mapped to the respective regions of the reference genome.
(DOC)

S1 Table Primer sequences used in this study. (DOC)

S2 Table Summary of RNA-Seq data from leaves of the apple cultivar ‘Gala’. (DOC)

S3 Table Flavonoid biosynthesis pathway genes in the KEGG (Kyoto Encyclopedia of Genes and Genomes) analysis. (DOC)

S4 Table List of genes from the ‘Darkmagenta’ module. (DOC)

S5 Table List of genes from the ‘Darkorange’ module. (DOC)
