# Supplementary material for: Identification of new regulators through transcriptome analysis that regulate anthocyanin biosynthesis in apple leaves at low temperatures
Source: PLoS One. 2019 Jan 29;14(1):e0210672. doi: 10.1371/journal.pone.0210672 (PMC6350969; doi:10.1371/journal.pone.0210672)
Supplement: S1 Fig — Heat map of the correlations between biological replicates. The PCC (Pearson correlation coefficient) values are quantitative indicators of relative expression levels of all genes in each sample. (DOC) [file pone.0210672.s001.doc]

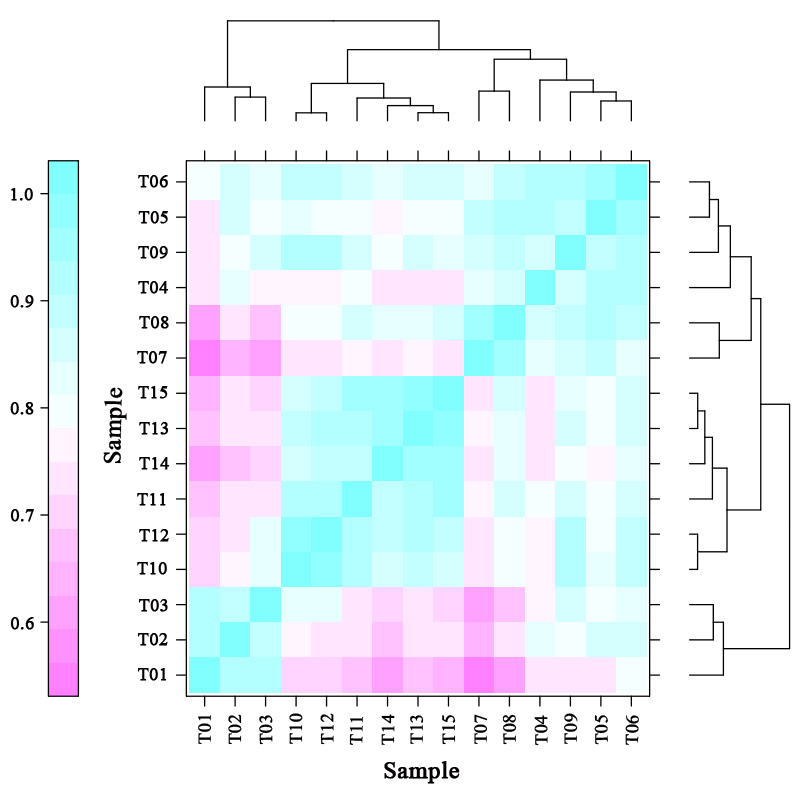


**Supplemental 1 Fig. Pearson correlation analysis.** Heat map of the correlations between biological replicates. The PCC (Pearson correlation coefficient) values are quantitative indicators of relative expression levels of all genes in each sample.
