# Supplementary material for: Identification of new regulators through transcriptome analysis that regulate anthocyanin biosynthesis in apple leaves at low temperatures
Source: PLoS One. 2019 Jan 29;14(1):e0210672. doi: 10.1371/journal.pone.0210672 (PMC6350969; doi:10.1371/journal.pone.0210672)
Supplement: S2 Fig — The terms exon, intron and intergenic refer to the percentage of cleaned reads mapped to the respective regions of the reference genome. (DOC) [file pone.0210672.s002.doc]

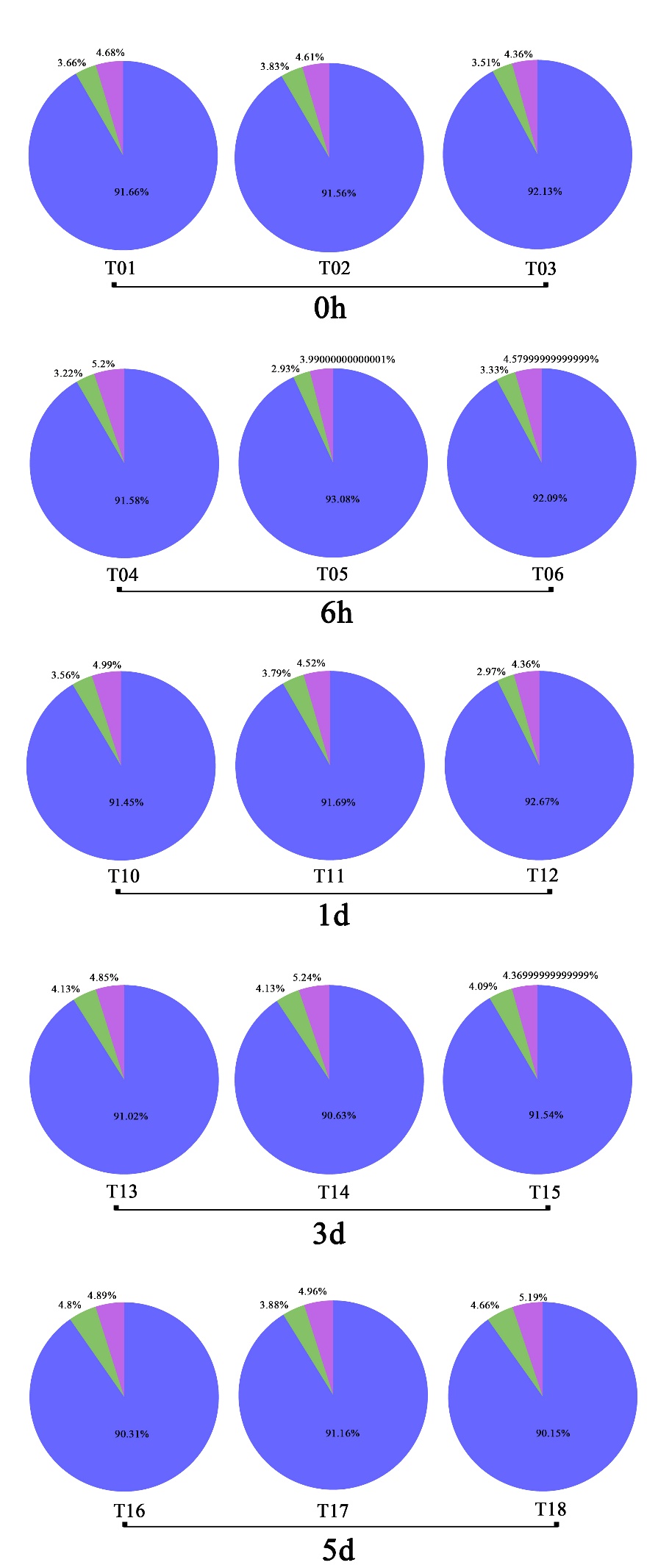


**Supplemental 2 Fig. The distribution of cleaned reads mapped to the reference genome.** The terms exon, intron and intergenic refer to the percentage of cleaned reads mapped to the respective regions of the reference genome.
