# Supplementary material for: Identification of new regulators through transcriptome analysis that regulate anthocyanin biosynthesis in apple leaves at low temperatures
Source: PLoS One. 2019 Jan 29;14(1):e0210672. doi: 10.1371/journal.pone.0210672 (PMC6350969; doi:10.1371/journal.pone.0210672)
Supplement: S1 Table — (DOC) [file pone.0210672.s003.doc]

**Supporting information**

**Supplemental 1 Table. Primer sequences used in this study.**

| **Gene ID** |  | **Sequences (5'-3')** |
| --- | --- | --- |
| DQ341382(18S) | F | GTCACTACCTCCCCGTGTCA |
|  | R | GAGCCTGAGAAACGGCTACC |
| MD04G1096200 (MdPAL) | F | ACCCTGGACAGATTGAGGCAGCT |
|  | R | GCGTAGCGATCCTGCTTTGGCT |
| MD13G1285100 (MdCHS) | F | GTGACTGTCCAGGAAGTTCGC |
|  | R | GCACACACTTGGATTCTCCTTTAG |
| MD15G1024100 (MdDFR) | F | GGACCCCGAGAATGAAGTG |
|  | R | CTCCACATTCACGGTTCCTG |
| MD02G1132200 (MdF3H) | F | TTCTACCCAAAATGCCCTCA |
|  | R | GTTTTCCCATCATCCCGTGT |
| MD03G1001100 (MdANS) | F | AGGGCCTGCATTGTTATCG |
|  | R | CTGCCCAGATGCATTGTTTG |
| MD07G1306900 (MdUFGT) | F | ACTAATGGTCAGCCCCATCT |
|  | R | ACGACAAGATCAAAGCGTCT |
| MD06G1211400 (MdLAR1) | F | TTTATCAAAGGATGCCAGGTT |
|  | R | CATCCAAGGTCCTGAAAGAAT |
| MD05G1335600 (MdANR1) | F | AACCACAAGAAGGTCTCCCAC |
|  | R | CCCTTGGATTGCTGGTTTGAT |
| MD15G1215500 (TT2/MdMYB12) | F | TTCCCAAGAAAGCAGGTTTGA |
|  | R | TGGCTGTGGGTTGATGAAAA |
| MD03G1297100（MdMYB22） | F | CGGACGCGGTTATAAGTGAT |
|  | R | CGTGATTTCCGAATCCACCT |
| MD17G1261000 (MdMYB114-like) | F | TACGGATCAATTCTCGCATGA |
|  | R | ACTCAAGACTGGGACATACAG |
| MD14G1031200 (MdLWD2-like) | F | GCACTGTCAGGGTGTTTGAT |
|  | R | GTCTCACCACATCCCAAATTAAAG |

F and R represents forward and reverse primers, respectively.
