# Supplementary material for: Identification of new regulators through transcriptome analysis that regulate anthocyanin biosynthesis in apple leaves at low temperatures
Source: PLoS One. 2019 Jan 29;14(1):e0210672. doi: 10.1371/journal.pone.0210672 (PMC6350969; doi:10.1371/journal.pone.0210672)
Supplement: S2 Table — (DOC) [file pone.0210672.s004.doc]

**Supplemental 2 Table. Summary of RNA-Seq data from leaves of the apple cultivar ‘Gala’.**

| **Sample name** | **Read**  **Sum** | **Clean reads** | **Clean**  **Bases** | **GC**  **(%)** | **Q20**  **(%)** | **Q30**  **(%)** | **Mapped**  **Reads** |
| --- | --- | --- | --- | --- | --- | --- | --- |
| **0h_1** | **21,191,893** | **21,191,893** | **6,336,524,248** | **46.86** | **97.46** | **93.2** | **38,382,796 (90.56%)** |
| **0h_2** | **24,871,144** | **24,871,144** | **7,428,882,020** | **47.02** | **96.95** | **92.5** | **44,324,010 (89.11%)** |
| **0h_3** | **24,524,356** | **24,524,356** | **7,327,194,018** | **47.14** | **96.92** | **92.43** | **43,767,628 (89.23%)** |
| **6h_1** | **24,820,821** | **24,820,821** | **7,407,209,564** | **47.49** | **97.06** | **92.71** | **44,427,852 (89.50%)** |
| **6h_2** | **24,502,162** | **24,502,162** | **7,315,717,116** | **47.22** | **96.89** | **92.49** | **43,666,539 (89.11%)** |
| **6h_3** | **28,386,947** | **28,386,947** | **8,472,386,670** | **47.2** | **97.11** | **92.81** | **50,695,763 (89.29%)** |
| **1d_1** | **20,724,414** | **20,724,414** | **6,200,878,680** | **46.76** | **97.53** | **93.37** | **37,154,154 (89.64%)** |
| **1d_2** | **29,792,989** | **29,792,989** | **8,884,841,926** | **47** | **97.26** | **93.07** | **53,488,438 (89.77%)** |
| **1d_3** | **30,185,967** | **26,584,537** | **8,996,281,886** | **46.84** | **96.77** | **92.09** | **53,794,519 (89.11%)** |
| **3d_1** | **26,584,537** | **26,584,537** | **7,934,293,158** | **46.65** | **96.95** | **92.5** | **47,229,596 (88.83%)** |
| **3d_1** | **25,474,071** | **25,474,071** | **7,604,304,478** | **46.69** | **97.07** | **92.77** | **45,337,565 (88.99%)** |
| **3d_1** | **33,938,202** | **33,938,202** | **10,109,998,470** | **46.79** | **97.21** | **92.94** | **60,768,297 (89.53%)** |
| **5d_1** | **22,474,828** | **22,474,828** | **6,711,791,288** | **46.65** | **97.2** | **92.98** | **40,232,672 (89.51%)** |
| **5d_2** | **24,748,208** | **24,748,208** | **7,391,548,404** | **46.99** | **96.87** | **92.45** | **43,883,770 (88.66%)** |
| **5d_3** | **24,961,374** | **24,961,374** | **7,449,783,922** | **46.49** | **97.07** | **92.61** | **44,419,270 (88.98%)** |
