# Supplementary material for: Identification of new regulators through transcriptome analysis that regulate anthocyanin biosynthesis in apple leaves at low temperatures
Source: PLoS One. 2019 Jan 29;14(1):e0210672. doi: 10.1371/journal.pone.0210672 (PMC6350969; doi:10.1371/journal.pone.0210672)
Supplement: S3 Table — (DOC) [file pone.0210672.s005.doc]

**Supplemental 3 Table. Flavonoid biosynthesis pathway genes in the KEGG (Kyoto Encyclopedia of Genes and Genomes) analysis.**

| **gene name** | **gene ID** |  |
| --- | --- | --- |
| Naringenin,2-oxoglutarate 3-dioxygenase | MD02G1132200 | 0h&6h; 0h&1d; 0h&3d; 0h&5d |
| Leucoanthocyanidin dioxygenase | MD03G1001100 | 0h&6h; 0h&1d; 0h&3d; 0h&5d |
| Polyketide synthase 5 | MD04G1003300 | 0h&6h; 0h&1d; 0h&3d; 0h&5d |
| Polyketide synthase 1 | MD04G1003400 | 0h&6h; 0h&1d; 0h&3d; 0h&5d |
| Leucoanthocyanidin dioxygenase | MD06G1071600 | 0h&6h; 0h&1d; 0h&3d; 0h&5d |
| Chalcone--flavonone isomerase | MD07G1186300 | 0h&6h; 0h&1d; 0h&3d; 0h&5d |
| flavanone 4-reductase | MD08G1028600 | 0h&6h; 0h&1d; 0h&3d; 0h&5d |
| Flavonol synthase | MD08G1121600 | 0h&6h; 0h&1d; 0h&3d; 0h&5d |
| Flavonol synthase | MD08G1168600 | 0h&6h; 0h&1d; 0h&3d; 0h&5d |
| Flavonoid 3&apos | MD14G1210700 | 0h&6h; 0h&1d; 0h&3d; 0h&5d |
| flavanone 4-reductase | MD15G1024100 | 0h&6h; 0h&1d; 0h&3d; 0h&5d |
| Naringenin,2-oxoglutarate 3-dioxygenase | MD15G1246200 | 0h&6h; 0h&1d; 0h&3d; 0h&5d |
| Flavonoid 3&apos | MD06G1201700 | 0h&6h; 0h&3d; 0h&5d |
| Leucoanthocyanidin reductase | MD06G1211400 | 0h&6h; 0h&3d; 0h&5d |
| Cytochrome P450 98A2 | MD08G1242900 | 0h&6h; 0h&1d; 0h&5d |
| Polyketide synthase 5 | MD04G1003000 | 0h&1d; 0h&3d; 0h&5d |
| Anthocyanidin reductase | MD05G1335600 | 0h&1d; 0h&3d; 0h&5d |
| Polyketide synthase | MD13G1285100 | 0h&1d; 0h&3d; 0h&5d |
| Cytochrome P450 98A2 | MD08G1243000 | 0h&6h; 0h&5d |
| Salutaridinol 7-O-acetyltransferase | MD10G1199300 | 0h&3d; 0h&5d |
| Anthocyanidin reductase | MD10G1311100 | 0h&3d; 0h&5d |
| Trans-cinnamate 4-monooxygenase | MD11G1052900 | 0h&3d; 0h&5d |
| Leucoanthocyanidin reductase | MD13G1046900 | 0h&3d; 0h&5d |
| Flavonol synthase | MD15G1353800 | 0h&3d; 0h&5d |
| Trans-cinnamate 4-monooxygenase | MD00G1221400 | 0h&3d |
| Shikimate O-hydroxycinnamoyltransferase | MD04G1188000 | 0h&3d |
| Cytochrome P450 98A2 | MD08G1243000 | 0h&5d |
| 3,5-dihydroxybiphenyl synthase | MD15G1132000 | 0h&5d |
| Cytochrome P450 98A2 | MD15G1436500 | 0h&5d |
| Leucoanthocyanidin reductase | MD16G1048500 | 0h&5d |
