# Supplementary material for: Identification of new regulators through transcriptome analysis that regulate anthocyanin biosynthesis in apple leaves at low temperatures
Source: PLoS One. 2019 Jan 29;14(1):e0210672. doi: 10.1371/journal.pone.0210672 (PMC6350969; doi:10.1371/journal.pone.0210672)
Supplement: S4 Table — (DOC) [file pone.0210672.s006.doc]

**Supplemental 4 Table. List of genes from the ‘Darkmagenta’ module.**

|  | **Gene ID** | **Blast swiss prot annotation** |
| --- | --- | --- |
| **Darkmagenta** | MD00G1038700 | -- |
|  | MD00G1073200 | Protein BIC1 OS=*Arabidopsis thaliana* GN=BIC1 PE=1 SV=1 |
|  | MD00G1140100 | Uncharacterized protein ycf68 OS=*Saccharum officinarum* GN=ycf68-1 PE=3 SV=1 |
|  | MD00G1187100 | 2-methylene-furan-3-one reductase OS=*Fragaria vesca* GN=EO PE=2 SV=2 |
|  | MD01G1000200 | Amino-acid permease BAT1 homolog OS=*Oryza sativa* subsp. japonica GN=BAT1 PE=2 SV=1 |
|  | MD01G1163900 | Adenine/guanine permease AZG1 OS=*Arabidopsis thaliana* GN=AZG1 PE=2 SV=1 |
|  | MD01G1233900 | -- |
|  | MD02G1034900 | Dynein 8 kDa light chain, flagellar outer arm OS=*Chlamydomonas reinhardtii* PE=1 SV=1 |
|  | MD02G1049600 | Ribulose bisphosphate carboxylase large chain OS=*Lotus* *japonicus* GN=rbcL PE=3 SV=1 |
|  | MD02G1068900 | ATP-dependent 6-phosphofructokinase 3 OS=*Arabidopsis thaliana* GN=PFK3 PE=1 SV=1 |
|  | MD02G1095700 | Cellulose synthase-like protein E2 OS=*Oryza sativa* subsp. japonica GN=CSLE2 PE=2 SV=1 |
|  | MD02G1099400 | Photosystem I P700 chlorophyll a apoprotein A1 OS=*Populus trichocarpa* GN=psaA PE=3 SV=1 |
|  | MD02G1170900 | -- |
|  | MD02G1179300 | -- |
|  | MD02G1261000 | Steroid 5-alpha-reductase DET2 OS=*Gossypium hirsutum* GN=DET2 PE=1 SV=1 |
|  | MD02G1272300 | -- |
|  | MD02G1292100 | Shewanella-like protein phosphatase 2 OS=*Arabidopsis thaliana* GN=SLP2 PE=1 SV=1 |
|  | MD02G1292200 | -- |
|  | MD02G1292400 | -- |
|  | MD03G1005600 | IQ domain-containing protein IQM2 OS=*Arabidopsis thaliana* GN=IQM2 PE=2 SV=1 |
|  | MD03G1099300 | Zinc finger protein ZAT12 OS=*Arabidopsis thaliana* GN=ZAT12 PE=2 SV=1 |
|  | MD03G1128400 | Probable histone H2B.3 OS=*Medicago truncatula* PE=3 SV=3 |
|  | MD03G1175000 | Chaperonin-like RbcX protein 2, chloroplastic OS=*Arabidopsis thaliana* GN=RBCX2 PE=1 SV=1 |
|  | MD03G1190300 | Pirin-like protein OS=*Solanum lycopersicum* PE=2 SV=1 |
|  | MD03G1194500 | -- |
|  | MD04G1112100 | Glutathione S-transferase L3 OS=*Arabidopsis thaliana* GN=GSTL3 PE=2 SV=1 |
|  | MD04G1142900 | Laccase-7 OS=*Arabidopsis thaliana* GN=LAC7 PE=2 SV=1 |
|  | MD04G1154600 | Trihelix transcription factor GT-3a OS=*Arabidopsis thaliana* GN=GT-3A PE=1 SV=1 |
|  | MD04G1161900 | -- |
|  | MD04G1216200 | -- |
|  | MD04G1244500 | Probable tyrosine-protein phosphatase At1g05000 OS=*Arabidopsis thaliana* GN=At1g05000 PE=1 SV=1 |
|  | MD05G1105500 | Phytosulfokines OS=*Asparagus officinalis* GN=PSK PE=1 SV=1 |
|  | MD05G1116400 | Proline-rich protein 4 OS=*Arabidopsis thaliana* GN=PRP4 PE=2 SV=1 |
|  | MD05G1178100 | Protein SULFUR DEFICIENCY-INDUCED 1 OS=*Arabidopsis thaliana* GN=SDI1 PE=2 SV=1 |
|  | MD05G1184200 | Probable glutathione S-transferase OS=*Nicotiana tabacum* PE=2 SV=1 |
|  | MD05G1196800 | Legumin A OS=*Gossypium hirsutum* GN=LEGA PE=2 SV=2 |
|  | MD05G1300200 | Abscisic acid receptor PYL6 OS=*Arabidopsis thaliana* GN=PYL6 PE=1 SV=1 |
|  | MD05G1305700 | Glutamate receptor 2.7 OS=*Arabidopsis thaliana* GN=GLR2.7 PE=2 SV=3 |
|  | MD05G1305900 | Glutamate receptor 2.7 OS=*Arabidopsis thaliana* GN=GLR2.7 PE=2 SV=3 |
|  | MD05G1325700 | External alternative NAD(P)H-ubiquinone oxidoreductase B2, mitochondrial OS=*Arabidopsis thaliana* GN=NDB2 PE=1 SV=1 |
|  | MD05G1356900 | -- |
|  | MD06G1110900 | 3-oxoacyl-[acyl-carrier-protein] synthase I, chloroplastic OS=*Arabidopsis thaliana* GN=KAS1 PE=1 SV=2 |
|  | MD06G1132900 | Acyl carrier protein 1, chloroplastic OS=*Casuarina glauca* GN=ACP1 PE=2 SV=1 |
|  | MD06G1134100 | Early light-induced protein 1, chloroplastic OS=*Arabidopsis thaliana* GN=ELIP1 PE=1 SV=1 |
|  | MD06G1160900 | Peptide methionine sulfoxide reductase OS=*Fragaria ananassa* PE=2 SV=2 |
|  | MD06G1212600 | -- |
|  | MD06G1224000 | Probable S-adenosylmethionine-dependent methyltransferase At5g38780 OS=*Arabidopsis thaliana* GN=At5g38780 PE=2 SV=1 |
|  | MD07G1009400 | -- |
|  | MD07G1192900 | Zinc finger protein ZAT11 OS=*Arabidopsis thaliana* GN=ZAT11 PE=2 SV=1 |
|  | MD07G1248300 | Oleosin 1 OS=*Prunus dulcis* GN=OLE1 PE=2 SV=1 |
|  | MD07G1303600 | -- |
|  | MD07G1305600 | Cell division cycle 20.1, cofactor of APC complex OS=*Arabidopsis thaliana* GN=CDC20-1 PE=1 SV=1 |
|  | MD07G1308000 | Laccase-15 OS=*Arabidopsis thaliana* GN=TT10 PE=1 SV=1 |
|  | MD08G1009800 | GDSL esterase/lipase EXL3 OS=*Arabidopsis thaliana* GN=EXL3 PE=2 SV=1 |
|  | MD08G1029500 | Isoflavone reductase-like protein OS=*Olea europaea* PE=1 SV=1 |
|  | MD08G1029600 | Isoflavone reductase-like protein OS=*Olea europaea* PE=1 SV=1 |
|  | MD08G1032800 | Cytochrome P450 734A1 OS=*Arabidopsis thaliana* GN=CYP734A1 PE=2 SV=1 |
|  | MD08G1038300 | -- |
|  | MD08G1121600 | Flavonol synthase/flavanone 3-hydroxylase OS=*Malus* *domestica* GN=FLS PE=2 SV=1 |
|  | MD08G1124700 | Gamma-glutamylcyclotransferase 2-2 OS=*Arabidopsis thaliana* GN=GGCT2;2 PE=1 SV=1 |
|  | MD08G1141400 | Pleiotropic drug resistance protein 1 OS=*Nicotiana tabacum* GN=PDR1 PE=2 SV=1 |
|  | MD08G1175700 | -- |
|  | MD09G1033200 | 2-methylene-furan-3-one reductase OS=*Fragaria vesca* GN=EO PE=2 SV=2 |
|  | MD09G1033600 | 2-methylene-furan-3-one reductase OS=*Fragaria ananassa* GN=EO PE=1 SV=2 |
|  | MD09G1034000 | 2-methylene-furan-3-one reductase OS=*Fragaria vesca* GN=EO PE=2 SV=2 |
|  | MD09G1036100 | -- |
|  | MD09G1057200 | Cell division topological specificity factor homolog, chloroplastic OS=*Arabidopsis thaliana* GN=MINE1 PE=1 SV=1 |
|  | MD09G1074100 | Agamous-like MADS-box protein AGL8 homolog OS=*Solanum lycopersicum* GN=TDR4 PE=2 SV=1 |
|  | MD09G1104900 | Cyclin-U1-1 OS=*Arabidopsis thaliana* GN=CYCU1-1 PE=1 SV=1 |
|  | MD09G1110300 | 5&apos;-adenylylsulfate reductase 1, chloroplastic OS=*Arabidopsis thaliana* GN=APR1 PE=1 SV=2 |
|  | MD09G1115500 | Triacylglycerol lipase 2 OS=*Arabidopsis thaliana* GN=LIP2 PE=2 SV=1 |
|  | MD09G1146100 | Uncharacterized oxidoreductase At1g06690, chloroplastic OS=*Arabidopsis thaliana* GN=At1g06690 PE=1 SV=1 |
|  | MD09G1169800 | -- |
|  | MD09G1202600 | Clavaminate synthase-like protein At3g21360 OS=*Arabidopsis thaliana* GN=At3g21360 PE=1 SV=1 |
|  | MD09G1233300 | Protein trichome birefringence-like 43 OS=*Arabidopsis* *thaliana* GN=TBL43 PE=2 SV=1 |
|  | MD09G1240700 | E3 ubiquitin-protein ligase ATL41 OS=*Arabidopsis thaliana* GN=ATL41 PE=1 SV=1 |
|  | MD09G1242800 | DNA-directed RNA polymerases II, IV and V subunit 6A OS=*Arabidopsis thaliana* GN=NRPB6A PE=1 SV=1 |
|  | MD10G1066100 | -- |
|  | MD10G1068300 | Protein translation factor SUI1 homolog 1 OS=*Arabidopsis* *thaliana* GN=At4g27130 PE=1 SV=2 |
|  | MD10G1073500 | Isoflavone reductase homolog OS=*Solanum tuberosum* PE=2 SV=1 |
|  | MD10G1085800 | Allene oxide synthase 3 OS=*Solanum lycopersicum* GN=AOS3 PE=1 SV=1 |
|  | MD10G1088100 | Protein DMR6-LIKE OXYGENASE 2 OS=*Arabidopsis thaliana* GN=DLO2 PE=2 SV=1 |
|  | MD10G1118600 | Proline-rich protein 4 OS=*Arabidopsis thaliana* GN=PRP4 PE=2 SV=1 |
|  | MD10G1142200 | Mitochondrial phosphate carrier protein 1, mitochondrial OS=*Arabidopsis thaliana* GN=MPT1 PE=2 SV=1 |
|  | MD10G1166300 | Protein SULFUR DEFICIENCY-INDUCED 1 OS=*Arabidopsis thaliana* GN=SDI1 PE=2 SV=1 |
|  | MD10G1171100 | GDSL esterase/lipase At5g33370 OS=*Arabidopsis thaliana* GN=At5g33370 PE=2 SV=1 |
|  | MD10G1172100 | Probable glutathione S-transferase OS=*Nicotiana tabacum* PE=2 SV=1 |
|  | MD10G1192000 | Probable galacturonosyltransferase 6 OS=*Arabidopsis thaliana* GN=GAUT6 PE=2 SV=1 |
|  | MD10G1198300 | Photosystem II 10 kDa polypeptide, chloroplastic OS=*Nicotiana tabacum* GN=PSBR PE=2 SV=1 |
|  | MD10G1301300 | Internal alternative NAD(P)H-ubiquinone oxidoreductase A2, mitochondrial OS=*Arabidopsis thaliana* GN=NDA2 PE=2 SV=1 |
|  | MD10G1314500 | Gibberellin 2-beta-dioxygenase 8 OS=*Arabidopsis thaliana* GN=GA2OX8 PE=1 SV=2 |
|  | MD10G1330600 | -- |
|  | MD11G1068500 | Photosystem I P700 chlorophyll a apoprotein A2 OS=*Buxus* *microphylla* GN=psaB PE=3 SV=1 |
|  | MD11G1215200 | 5&apos;-adenylylsulfate reductase 3, chloroplastic OS=*Arabidopsis thaliana* GN=APR3 PE=2 SV=2 |
|  | MD11G1305600 | -- |
|  | MD12G1035800 | Protein BIC1 OS=*Arabidopsis thaliana* GN=BIC1 PE=1 SV=1 |
|  | MD12G1157300 | -- |
|  | MD12G1161200 | -- |
|  | MD12G1162100 | Glycerol-3-phosphate 2-O-acyltransferase 6 OS=*Arabidopsis* *thaliana* GN=GPAT6 PE=1 SV=1 |
|  | MD12G1179400 | Protein DETOXIFICATION 44, chloroplastic OS=*Arabidopsis thaliana* GN=DTX44 PE=2 SV=1 |
|  | MD12G1218000 | Probable linoleate 9S-lipoxygenase 5 OS=*Solanum tuberosum* GN=LOX1.5 PE=2 SV=1 |
|  | MD12G1252200 | GDSL esterase/lipase At5g33370 OS=*Arabidopsis thaliana* GN=At5g33370 PE=2 SV=1 |
|  | MD13G1003800 | Chaperone protein dnaJ 20, chloroplastic OS=*Arabidopsis thaliana* GN=ATJ20 PE=1 SV=2 |
|  | MD13G1042500 | Tetraketide alpha-pyrone reductase 2 OS=*Arabidopsis thaliana* GN=TKPR2 PE=1 SV=1 |
|  | MD13G1045600 | -- |
|  | MD13G1064000 | -- |
|  | MD13G1076200 | Probable serine/threonine-protein kinase PBL3 OS=*Arabidopsis thaliana* GN=PBL3 PE=1 SV=1 |
|  | MD13G1095000 | -- |
|  | MD13G1200100 | Early light-induced protein 1, chloroplastic OS=*Arabidopsis* *thaliana* GN=ELIP1 PE=1 SV=1 |
|  | MD13G1200300 | Early light-induced protein 1, chloroplastic OS=*Arabidopsis thaliana* GN=ELIP1 PE=1 SV=1 |
|  | MD13G1200600 | Early light-induced protein 1, chloroplastic OS=*Arabidopsis thaliana* GN=ELIP1 PE=1 SV=1 |
|  | MD13G1231400 | Protein SULFUR DEFICIENCY-INDUCED 2 OS=*Arabidopsis thaliana* GN=At1g04770 PE=2 SV=1 |
|  | MD14G1014500 | Peroxiredoxin-2E, chloroplastic OS=*Arabidopsis thaliana* GN=PRXIIE PE=1 SV=2 |
|  | MD14G1090500 | Probable glucan 1,3-alpha-glucosidase OS=*Arabidopsis thaliana* GN=PSL5 PE=1 SV=1 |
|  | MD14G1150400 | Early light-induced protein, chloroplastic OS=*Pisum sativum* PE=2 SV=1 |
|  | MD14G1165800 | CBBY-like protein OS=*Arabidopsis thaliana* GN=CBBY PE=1 SV=1 |
|  | MD14G1204300 | Protein NEN1 OS=*Arabidopsis thaliana* GN=NEN1 PE=2 SV=1 |
|  | MD14G1207600 | Probable 2-oxoglutarate-dependent dioxygenase At5g05600 OS=*Arabidopsis thaliana* GN=At5g05600 PE=2 SV=1 |
|  | MD14G1239800 | U-box domain-containing protein 21 OS=*Arabidopsis thaliana* GN=PUB21 PE=2 SV=1 |
|  | MD15G1037300 | -- |
|  | MD15G1104100 | Gamma-glutamylcyclotransferase 2-2 OS=*Arabidopsis thaliana* GN=GGCT2;2 PE=1 SV=1 |
|  | MD15G1133000 | Probable calcium-binding protein CML44 OS=*Arabidopsis thaliana* GN=CML44 PE=2 SV=2 |
|  | MD15G1134000 | ATP synthase subunit c, chloroplastic OS=*Atropa belladonna* GN=atpH PE=3 SV=1 |
|  | MD15G1191300 | Bark storage protein A OS=*Populus deltoides* GN=BSPA PE=2 SV=1 |
|  | MD15G1328900 | L-ascorbate oxidase OS=*Cucumis sativus* PE=1 SV=1 |
|  | MD15G1346300 | -- |
|  | MD15G1360000 | Serine hydroxymethyltransferase 7 OS=*Arabidopsis thaliana* GN=SHM7 PE=2 SV=1 |
|  | MD15G1384500 | MADS-box protein AGL24 OS=*Arabidopsis thaliana* GN=AGL24 PE=1 SV=1 |
|  | MD15G1402700 | 12-oxophytodienoate reductase 2 OS=*Arabidopsis thaliana* GN=OPR2 PE=1 SV=2 |
|  | MD15G1415100 | Cellulose synthase-like protein G2 OS=*Arabidopsis thaliana* GN=CSLG2 PE=2 SV=1 |
|  | MD15G1434600 | -- |
|  | MD16G1011600 | Glutathione transferase GST 23 OS=*Zea mays* PE=2 SV=1 |
|  | MD16G1043200 | Homocysteine S-methyltransferase 1 OS=*Brassica oleracea* var. italica GN=HMT1 PE=1 SV=1 |
|  | MD16G1064700 | -- |
|  | MD16G1065700 | Chorismate mutase 3, chloroplastic OS=*Arabidopsis thaliana* GN=CM3 PE=1 SV=1 |
|  | MD16G1080300 | Thioredoxin H2 OS=*Arabidopsis thaliana* GN=TRX2 PE=2 SV=2 |
|  | MD16G1080400 | Thioredoxin H2 OS=*Arabidopsis thaliana* GN=TRX2 PE=2 SV=2 |
|  | MD16G1088600 | MLP-like protein 423 OS=*Arabidopsis thaliana* GN=MLP423 PE=2 SV=1 |
|  | MD16G1096100 | -- |
|  | MD16G1128500 | -- |
|  | MD16G1160600 | Major allergen Pru ar 1 OS=*Prunus armeniaca* PE=1 SV=1 |
|  | MD16G1283100 | PsbP domain-containing protein 3, chloroplastic OS=*Arabidopsis thaliana* GN=PPD3 PE=1 SV=2 |
|  | MD17G1019100 | -- |
|  | MD17G1028200 | -- |
|  | MD17G1075600 | 30S ribosomal protein 3, chloroplastic OS=*Spinacia oleracea* GN=PSRP3 PE=1 SV=1 |
|  | MD17G1079900 | -- |
|  | MD17G1102000 | Ribonuclease 3-like protein 3 OS=*Oryza sativa* subsp. japonica GN=Os06g0358800 PE=2 SV=1 |
|  | MD17G1154800 | RING-H2 finger protein ATL60 OS=*Arabidopsis thaliana* GN=ATL60 PE=2 SV=1 |
|  | MD17G1245500 | -- |
|  | MD17G1284300 | ABC transporter B family member 21 OS=*Arabidopsis* *thaliana* GN=ABCB21 PE=1 SV=2 |
|  | Malus_domestica_newGene_10218 | -- |
|  | Malus_domestica_newGene_3491 | -- |
