# Supplementary material for: Identification of new regulators through transcriptome analysis that regulate anthocyanin biosynthesis in apple leaves at low temperatures
Source: PLoS One. 2019 Jan 29;14(1):e0210672. doi: 10.1371/journal.pone.0210672 (PMC6350969; doi:10.1371/journal.pone.0210672)
Supplement: S5 Table — (DOC) [file pone.0210672.s007.doc]

**Supplemental 5 Table. List of genes from the ‘Darkorange’ module.**

|  | **Gene ID** | **Blast swiss prot annotation** |
| --- | --- | --- |
| **Darkorange** | MD00G1025900 | -- |
|  | MD00G1037100 | Phospho-2-dehydro-3-deoxyheptonate aldolase 1, chloroplastic OS=*Nicotiana tabacum* GN=DHAPS-1 PE=2 SV=1 |
|  | MD00G1067800 | -- |
|  | MD00G1072500 | Cinnamoyl-CoA reductase-like SNL6 OS=*Oryza sativa* subsp. japonica GN=SNL6 PE=3 SV=1 |
|  | MD00G1204600 | Cytochrome P450 78A6 OS=*Arabidopsis thaliana* GN=CYP78A6 PE=2 SV=1 |
|  | MD01G1017100 | Transcription factor MYB113 OS=*Arabidopsis thaliana* GN=MYB113 PE=1 SV=1 |
|  | MD01G1076200 | -- |
|  | MD01G1167300 | Probable chalcone--flavonone isomerase 3 OS=*Arabidopsis thaliana* GN=CHI3 PE=1 SV=1 |
|  | MD01G1234400 | Anthocyanidin 3-O-glucosyltransferase 2 OS=*Fragaria ananassa* GN=FGT PE=1 SV=1 |
|  | MD01G1236300 | 4-coumarate--CoA ligase 2 OS=*Glycine max* PE=2 SV=2 |
|  | MD02G1034100 | Adenine nucleotide transporter BT1, chloroplastic/mitochondrial OS=*Arabidopsis thaliana* GN=BT1 PE=1 SV=1 |
|  | MD02G1034900 | Dynein 8 kDa light chain, flagellar outer arm OS=*Chlamydomonas reinhardtii* PE=1 SV=1 |
|  | MD02G1044800 | -- |
|  | MD02G1118000 | Dehydrodolichyl diphosphate synthase 6 OS=*Arabidopsis* *thaliana* GN=At2g17570 PE=2 SV=2 |
|  | MD02G1132200 | Naringenin,2-oxoglutarate 3-dioxygenase OS=*Malus* *domestica* PE=2 SV=1 |
|  | MD02G1144500 | Pheophytinase, chloroplastic OS=*Arabidopsis thaliana* GN=PPH PE=1 SV=1 |
|  | MD02G1158500 | -- |
|  | MD02G1172500 | -- |
|  | MD02G1261000 | Steroid 5-alpha-reductase DET2 OS=*Gossypium hirsutum* GN=DET2 PE=1 SV=1 |
|  | MD02G1272300 | -- |
|  | MD02G1292400 | -- |
|  | MD02G1302400 | -- |
|  | MD03G1001100 | Leucoanthocyanidin dioxygenase OS=*Malus domestica* GN=ANS PE=2 SV=1 |
|  | MD03G1058400 | Protein NUCLEAR FUSION DEFECTIVE 4 OS=*Arabidopsis thaliana* GN=NFD4 PE=3 SV=1 |
|  | MD03G1069500 | Isoflavonoid 7-O-beta-apiosyl-glucoside beta-glycosidase OS=*Dalbergia nigrescens* PE=1 SV=1 |
|  | MD03G1102500 | 3-ketoacyl-CoA synthase 12 OS=*Arabidopsis thaliana* GN=KCS12 PE=2 SV=1 |
|  | MD03G1175000 | Chaperonin-like RbcX protein 2, chloroplastic OS=*Arabidopsis thaliana* GN=RBCX2 PE=1 SV=1 |
|  | MD03G1215100 | -- |
|  | MD03G1294200 | Protein trichome birefringence-like 6 OS=*Arabidopsis* *thaliana* GN=TBL6 PE=2 SV=1 |
|  | MD03G1294300 | Protein trichome birefringence-like 6 OS=*Arabidopsis thaliana* GN=TBL6 PE=2 SV=1 |
|  | MD03G1297100 | Myb-related protein P OS=*Zea mays* GN=P PE=2 SV=1 |
|  | MD04G1003300 | Polyketide synthase 5 OS=*Rubus idaeus* GN=PKS5 PE=1 SV=1 |
|  | MD04G1003400 | Polyketide synthase 1 OS=*Rubus idaeus* GN=PKS1 PE=1 SV=1 |
|  | MD04G1052600 | Expansin-like B1 OS=*Arabidopsis thaliana* GN=EXLB1 PE=2 SV=2 |
|  | MD04G1069500 | Bifunctional dTDP-4-dehydrorhamnose 3,5-epimerase/dTDP-4-dehydrorhamnose reductase OS=*Arabidopsis thaliana* GN=NRS/ER PE=1 SV=1 |
|  | MD04G1096200 | Phenylalanine ammonia-lyase 1 OS=*Prunus avium* GN=PAL1 PE=2 SV=1 |
|  | MD04G1131000 | Probable WRKY transcription factor 49 OS=*Arabidopsis* *thaliana* GN=WRKY49 PE=2 SV=1 |
|  | MD04G1132400 | Protein SRG1 OS=*Arabidopsis thaliana* GN=SRG1 PE=2 SV=1 |
|  | MD04G1139100 | -- |
|  | MD04G1146900 | Probable anion transporter 3, chloroplastic OS=*Arabidopsis thaliana* GN=ANTR3 PE=2 SV=2 |
|  | MD04G1216200 | -- |
|  | MD04G1216300 | -- |
|  | MD04G1243200 | Probable glycosyltransferase At5g20260 OS=*Arabidopsis thaliana* GN=At5g20260 PE=3 SV=3 |
|  | MD04G1243300 | Probable glycosyltransferase At5g20260 OS=*Arabidopsis* *thaliana* GN=At5g20260 PE=3 SV=3 |
|  | MD05G1102900 | Serine carboxypeptidase-like 18 OS=*Arabidopsis thaliana* GN=SCPL18 PE=2 SV=2 |
|  | MD05G1103600 | Serine carboxypeptidase-like 18 OS=*Arabidopsis thaliana* GN=SCPL18 PE=2 SV=2 |
|  | MD05G1104400 | -- |
|  | MD05G1117500 | Dirigent protein 23 OS=*Arabidopsis thaliana* GN=DIR23 PE=2 SV=1 |
|  | MD05G1169500 | Cyclin-C1-2 OS=*Arabidopsis thaliana* GN=CYCC1-2 PE=2 SV=1 |
|  | MD05G1178100 | Protein SULFUR DEFICIENCY-INDUCED 1 OS=*Arabidopsis thaliana* GN=SDI1 PE=2 SV=1 |
|  | MD05G1215500 | -- |
|  | MD05G1269100 | -- |
|  | MD05G1276500 | Transcription factor MYB113 OS=*Arabidopsis thaliana* GN=MYB113 PE=1 SV=1 |
|  | MD05G1303900 | GDSL esterase/lipase At1g28600 OS=*Arabidopsis thaliana* GN=At1g28600 PE=2 SV=1 |
|  | MD05G1335600 | Anthocyanidin reductase ((2S)-flavan-3-ol-forming) OS=*Vitis* *vinifera* GN=ANR PE=3 SV=1 |
|  | MD05G1353200 | Pyrophosphate--fructose 6-phosphate 1-phosphotransferase subunit beta OS=*Ricinus communis* GN=PFP-BETA PE=3 SV=1 |
|  | MD06G1036600 | Cinnamoyl-CoA reductase-like SNL6 OS=*Oryza sativa* subsp. japonica GN=SNL6 PE=3 SV=1 |
|  | MD06G1053100 | Transcription repressor MYB5 OS=*Arabidopsis thaliana* GN=MYB5 PE=1 SV=1 |
|  | MD06G1071600 | Leucoanthocyanidin dioxygenase OS=*Malus domestica* GN=ANS PE=2 SV=1 |
|  | MD06G1088600 | Phosphoglycerate mutase-like protein AT74 OS=*Arabidopsis* *thaliana* GN=At3g05170 PE=2 SV=1 |
|  | MD06G1110900 | 3-oxoacyl-[acyl-carrier-protein] synthase I, chloroplastic OS=*Arabidopsis thaliana* GN=KAS1 PE=1 SV=2 |
|  | MD06G1112200 | -- |
|  | MD06G1112300 | -- |
|  | MD06G1197800 | Peroxisome biogenesis factor 10 OS=*Arabidopsis thaliana* GN=PEX10 PE=1 SV=1 |
|  | MD06G1201700 | Flavonoid 3&apos;-monooxygenase OS=*Petunia hybrida* GN=CYP75B2 PE=2 SV=1 |
|  | MD06G1211400 | Leucoanthocyanidin reductase OS=*Desmodium uncinatum* GN=LAR PE=1 SV=1 |
|  | MD06G1229600 | Myb-related protein 308 OS=*Antirrhinum majus* GN=MYB308 PE=2 SV=1 |
|  | MD06G1229700 | Myb-related protein 308 OS=*Antirrhinum majus* GN=MYB308 PE=2 SV=1 |
|  | MD06G1232300 | -- |
|  | MD07G1113400 | Cyclin-U2-1 OS=*Arabidopsis thaliana* GN=CYCU2-1 PE=1 SV=1 |
|  | MD07G1116600 | Putative lipid-binding protein At4g00165 OS=*Arabidopsis* *thaliana* GN=At4g00165 PE=2 SV=1 |
|  | MD07G1139000 | UPF0187 protein At3g61320, chloroplastic OS=*Arabidopsis thaliana* GN=At3g61320 PE=2 SV=2 |
|  | MD07G1145300 | Protein SUPPRESSOR OF PHYA-105 1 OS=*Arabidopsis thaliana* GN=SPA1 PE=1 SV=1 |
|  | MD07G1232800 | -- |
|  | MD07G1233400 | Probable chalcone--flavonone isomerase 3 OS=*Arabidopsis thaliana* GN=CHI3 PE=1 SV=1 |
|  | MD07G1266200 | Homeobox-leucine zipper protein ANTHOCYANINLESS 2 OS=*Arabidopsis thaliana* GN=ANL2 PE=2 SV=1 |
|  | MD07G1274200 | Short-chain dehydrogenase TIC 32, chloroplastic OS=*Pisum* *sativum* GN=TIC32 PE=1 SV=1 |
|  | MD07G1306900 | Anthocyanidin 3-O-glucosyltransferase 2 OS=*Fragaria ananassa* GN=FGT PE=1 SV=1 |
|  | MD07G1309000 | 4-coumarate--CoA ligase 2 OS=*Glycine max* PE=2 SV=2 |
|  | MD08G1002300 | 1,4-alpha-glucan-branching enzyme OS=*Solanum tuberosum* GN=SBE1 PE=2 SV=2 |
|  | MD08G1009800 | GDSL esterase/lipase EXL3 OS=*Arabidopsis thaliana* GN=EXL3 PE=2 SV=1 |
|  | MD08G1028600 | Bifunctional dihydroflavonol 4-reductase/flavanone 4-reductase OS=*Pyrus communis* GN=DFR PE=1 SV=1 |
|  | MD08G1070700 | Anthocyanin regulatory C1 protein OS=*Zea mays* GN=C1 PE=2 SV=1 |
|  | MD08G1087600 | Uncharacterized protein At2g23090 OS=*Arabidopsis thaliana* GN=At2g23090 PE=1 SV=1 |
|  | MD08G1121600 | Flavonol synthase/flavanone 3-hydroxylase OS=*Malus* *domestica* GN=FLS PE=2 SV=1 |
|  | MD08G1124700 | Gamma-glutamylcyclotransferase 2-2 OS=*Arabidopsis thaliana* GN=GGCT2;2 PE=1 SV=1 |
|  | MD08G1168600 | Flavonol synthase/flavanone 3-hydroxylase OS=*Malus domestica* GN=FLS PE=2 SV=1 |
|  | MD08G1178500 | -- |
|  | MD08G1178600 | -- |
|  | MD08G1192300 | Zinc-finger homeodomain protein 1 OS=*Oryza sativa* subsp. indica GN=ZHD1 PE=3 SV=1 |
|  | MD08G1249800 | -- |
|  | MD08G1250800 | Ferric reduction oxidase 7, chloroplastic OS=*Arabidopsis* *thaliana* GN=FRO7 PE=2 SV=1 |
|  | MD09G1032400 | Beta carbonic anhydrase 3 OS=*Arabidopsis thaliana* GN=BCA3 PE=2 SV=1 |
|  | MD09G1058000 | Chorismate mutase 1, chloroplastic OS=*Arabidopsis thaliana* GN=CM1 PE=1 SV=3 |
|  | MD09G1104900 | Cyclin-U1-1 OS=*Arabidopsis thaliana* GN=CYCU1-1 PE=1 SV=1 |
|  | MD09G1110400 | Aquaporin SIP1-1 OS=*Oryza sativa* subsp. japonica GN=SIP1-1 PE=2 SV=2 |
|  | MD09G1146100 | Uncharacterized oxidoreductase At1g06690, chloroplastic OS=*Arabidopsis thaliana* GN=At1g06690 PE=1 SV=1 |
|  | MD09G1146800 | Phytoene synthase, chloroplastic OS=*Daucus carota* GN=PSY PE=2 SV=1 |
|  | MD09G1149700 | -- |
|  | MD09G1169800 | -- |
|  | MD09G1183200 | Auxin-binding protein ABP19a OS=*Prunus persica* GN=ABP19A PE=3 SV=1 |
|  | MD09G1202600 | Clavaminate synthase-like protein At3g21360 OS=*Arabidopsis thaliana* GN=At3g21360 PE=1 SV=1 |
|  | MD09G1233300 | Protein trichome birefringence-like 43 OS=*Arabidopsis thaliana* GN=TBL43 PE=2 SV=1 |
|  | MD09G1246100 | -- |
|  | MD09G1252100 | Ribulose bisphosphate carboxylase small chain, chloroplastic OS=*Malus* sp. GN=RBCS PE=2 SV=1 |
|  | MD09G1270500 | -- |
|  | MD10G1012400 | PsbP domain-containing protein 4, chloroplastic OS=*Arabidopsis thaliana* GN=PPD4 PE=1 SV=2 |
|  | MD10G1059700 | Indole-3-acetic acid-induced protein ARG7 OS=*Vigna radiata* var. radiata GN=ARG7 PE=2 SV=1 |
|  | MD10G1062300 | NADP-dependent D-sorbitol-6-phosphate dehydrogenase OS=*Malus domestica* GN=S6PDH PE=2 SV=1 |
|  | MD10G1066100 | -- |
|  | MD10G1071000 | Salicylate carboxymethyltransferase OS=*Clarkia breweri* GN=SAMT PE=1 SV=1 |
|  | MD10G1088100 | Protein DMR6-LIKE OXYGENASE 2 OS=*Arabidopsis thaliana* GN=DLO2 PE=2 SV=1 |
|  | MD10G1089400 | La-related protein 6C OS=*Arabidopsis thaliana* GN=LARP6C PE=3 SV=1 |
|  | MD10G1118600 | Proline-rich protein 4 OS=*Arabidopsis thaliana* GN=PRP4 PE=2 SV=1 |
|  | MD10G1142400 | -- |
|  | MD10G1157200 | Chloroplast envelope quinone oxidoreductase homolog OS=*Arabidopsis thaliana* GN=CEQORH PE=1 SV=1 |
|  | MD10G1166300 | Protein SULFUR DEFICIENCY-INDUCED 1 OS=*Arabidopsis thaliana* GN=SDI1 PE=2 SV=1 |
|  | MD10G1171100 | GDSL esterase/lipase At5g33370 OS=*Arabidopsis thaliana* GN=At5g33370 PE=2 SV=1 |
|  | MD10G1198300 | Photosystem II 10 kDa polypeptide, chloroplastic OS=*Nicotiana tabacum* GN=PSBR PE=2 SV=1 |
|  | MD10G1249600 | -- |
|  | MD10G1283200 | GDSL esterase/lipase At1g28600 OS=*Arabidopsis thaliana* GN=At1g28600 PE=2 SV=1 |
|  | MD10G1297800 | -- |
|  | MD10G1303600 | External alternative NAD(P)H-ubiquinone oxidoreductase B2, mitochondrial OS=*Arabidopsis thaliana* GN=NDB2 PE=1 SV=1 |
|  | MD10G1311100 | Anthocyanidin reductase ((2S)-flavan-3-ol-forming) OS=*Vitis vinifera* GN=ANR PE=3 SV=1 |
|  | MD10G1314500 | Gibberellin 2-beta-dioxygenase 8 OS=*Arabidopsis thaliana* GN=GA2OX8 PE=1 SV=2 |
|  | MD10G1341600 | Probable galactinol--sucrose galactosyltransferase 2 OS=*Arabidopsis thaliana* GN=RFS2 PE=2 SV=2 |
|  | MD11G1009300 | Probable acyl-activating enzyme 18, peroxisomal OS=*Arabidopsis thaliana* GN=AAE18 PE=2 SV=1 |
|  | MD11G1052900 | Trans-cinnamate 4-monooxygenase OS=*Glycine max* GN=CYP73A11 PE=2 SV=1 |
|  | MD11G1064000 | Cinnamoyl-CoA reductase 1 OS=*Arabidopsis thaliana* GN=CCR1 PE=1 SV=1 |
|  | MD11G1079600 | Cytochrome P450 86A1 OS=*Arabidopsis thaliana* GN=CYP86A1 PE=1 SV=2 |
|  | MD11G1139600 | -- |
|  | MD11G1172600 | -- |
|  | MD11G1193100 | Chaperonin-like RbcX protein 2, chloroplastic OS=*Arabidopsis thaliana* GN=RBCX2 PE=1 SV=1 |
|  | MD11G1204800 | -- |
|  | MD11G1215200 | 5&apos;-adenylylsulfate reductase 3, chloroplastic OS=*Arabidopsis thaliana* GN=APR3 PE=2 SV=2 |
|  | MD11G1221200 | Fatty acid desaturase 4, chloroplastic OS=*Arabidopsis thaliana* GN=FAD4 PE=1 SV=1 |
|  | MD11G1240900 | Beta-glucosidase 18 OS=*Oryza sativa* subsp. japonica GN=BGLU18 PE=3 SV=2 |
|  | MD11G1242200 | -- |
|  | MD11G1268800 | Protein trichome birefringence-like 37 OS=*Arabidopsis thaliana* GN=TBL37 PE=2 SV=2 |
|  | MD11G1295700 | NAD(P)H-quinone oxidoreductase subunit K, chloroplastic OS=*Morus indica* GN=ndhK PE=3 SV=2 |
|  | MD11G1305600 | -- |
|  | MD11G1316800 | Myb-related protein P OS=*Zea mays* GN=P PE=2 SV=1 |
|  | MD12G1013100 | Probable lysophospholipase BODYGUARD 4 OS=*Arabidopsis thaliana* GN=BDG4 PE=2 SV=1 |
|  | MD12G1013500 | -- |
|  | MD12G1035800 | Protein BIC1 OS=*Arabidopsis thaliana* GN=BIC1 PE=1 SV=1 |
|  | MD12G1110000 | -- |
|  | MD12G1116700 | Phenylalanine ammonia-lyase 1 OS=*Prunus avium* GN=PAL1 PE=2 SV=1 |
|  | MD12G1119800 | -- |
|  | MD12G1128400 | Thioredoxin-like protein AAED1, chloroplastic OS=*Arabidopsis thaliana* GN=At2g37240 PE=1 SV=2 |
|  | MD12G1162100 | Glycerol-3-phosphate 2-O-acyltransferase 6 OS=*Arabidopsis thaliana* GN=GPAT6 PE=1 SV=1 |
|  | MD12G1179400 | Protein DETOXIFICATION 44, chloroplastic OS=*Arabidopsis thaliana* GN=DTX44 PE=2 SV=1 |
|  | MD12G1218200 | Probable linoleate 9S-lipoxygenase 5 OS=*Solanum tuberosum* GN=LOX1.5 PE=2 SV=1 |
|  | MD12G1262300 | Phosphoglycolate phosphatase 1B, chloroplastic OS=*Arabidopsis thaliana* GN=PGLP1B PE=1 SV=1 |
|  | MD13G1003800 | Chaperone protein dnaJ 20, chloroplastic OS=*Arabidopsis thaliana* GN=ATJ20 PE=1 SV=2 |
|  | MD13G1013800 | -- |
|  | MD13G1022400 | Protein C2-DOMAIN ABA-RELATED 9 OS=*Arabidopsis thaliana* GN=CAR9 PE=2 SV=1 |
|  | MD13G1025900 | -- |
|  | MD13G1042500 | Tetraketide alpha-pyrone reductase 2 OS=*Arabidopsis* *thaliana* GN=TKPR2 PE=1 SV=1 |
|  | MD13G1045600 | -- |
|  | MD13G1046900 | Leucoanthocyanidin reductase OS=*Desmodium uncinatum* GN=LAR PE=1 SV=1 |
|  | MD13G1064000 | -- |
|  | MD13G1095000 | -- |
|  | MD13G1186600 | Protein FANTASTIC FOUR 3 OS=*Arabidopsis thaliana* GN=FAF3 PE=2 SV=1 |
|  | MD13G1200600 | Early light-induced protein 1, chloroplastic OS=*Arabidopsis thaliana* GN=ELIP1 PE=1 SV=1 |
|  | MD13G1202900 | -- |
|  | MD13G1231400 | Protein SULFUR DEFICIENCY-INDUCED 2 OS=*Arabidopsis thaliana* GN=At1g04770 PE=2 SV=1 |
|  | MD13G1265500 | CBL-interacting serine/threonine-protein kinase 21 OS=*Arabidopsis thaliana* GN=CIPK21 PE=1 SV=1 |
|  | MD13G1285100 | Polyketide synthase 1 OS=*Rubus idaeus* GN=PKS1 PE=1 SV=1 |
|  | MD14G1014500 | Peroxiredoxin-2E, chloroplastic OS=*Arabidopsis thaliana* GN=PRXIIE PE=1 SV=2 |
|  | MD14G1020100 | -- |
|  | MD14G1021100 | Protein TERMINAL FLOWER 1 OS=*Arabidopsis thaliana* GN=TFL1 PE=1 SV=1 |
|  | MD14G1029500 | -- |
|  | MD14G1029600 | -- |
|  | MD14G1031200 | WD repeat-containing protein LWD2 OS=*Arabidopsis thaliana* GN=LWD2 PE=2 SV=1 |
|  | MD14G1081900 | Protein GAST1 OS=*Solanum lycopersicum* GN=GAST1 PE=2 SV=1 |
|  | MD14G1099500 | Protein LURP-one-related 3 OS=*Arabidopsis thaliana* GN=At1g53890 PE=2 SV=2 |
|  | MD14G1124200 | Oligopeptide transporter 7 OS=*Arabidopsis thaliana* GN=OPT7 PE=2 SV=1 |
|  | MD14G1137300 | Beta-carotene isomerase D27, chloroplastic OS=*Arabidopsis thaliana* GN=D27 PE=1 SV=1 |
|  | MD14G1146000 | -- |
|  | MD14G1165800 | CBBY-like protein OS=*Arabidopsis thaliana* GN=CBBY PE=1 SV=1 |
|  | MD14G1171300 | -- |
|  | MD14G1205300 | Peroxisome biogenesis factor 10 OS=*Arabidopsis thaliana* GN=PEX10 PE=1 SV=1 |
|  | MD14G1210700 | Flavonoid 3&apos;-monooxygenase OS=*Petunia hybrida* GN=CYP75B2 PE=2 SV=1 |
|  | MD14G1234500 | Transcription repressor MYB6 OS=*Arabidopsis thaliana* GN=MYB6 PE=1 SV=1 |
|  | MD14G1234600 | Myb-related protein 308 OS=*Antirrhinum majus* GN=MYB308 PE=2 SV=1 |
|  | MD15G1009500 | GDSL esterase/lipase EXL3 OS=*Arabidopsis thaliana* GN=EXL3 PE=2 SV=1 |
|  | MD15G1014100 | ATP sulfurylase 2 OS=*Arabidopsis thaliana* GN=APS2 PE=1 SV=1 |
|  | MD15G1024100 | Bifunctional dihydroflavonol 4-reductase/flavanone 4-reductase OS=*Malus domestica* GN=DFR PE=1 SV=1 |
|  | MD15G1051400 | Anthocyanin regulatory C1 protein OS=*Zea mays* GN=C1 PE=2 SV=1 |
|  | MD15G1096800 | Tetraspanin-19 OS=*Arabidopsis thaliana* GN=TOM2AH3 PE=2 SV=1 |
|  | MD15G1098800 | BURP domain protein RD22 OS=*Arabidopsis thaliana* GN=RD22 PE=2 SV=1 |
|  | MD15G1100300 | -- |
|  | MD15G1156700 | Receptor-like serine/threonine-protein kinase SD1-6 OS=*Arabidopsis thaliana* GN=SD16 PE=1 SV=2 |
|  | MD15G1188200 | -- |
|  | MD15G1215500 | Transcription factor TT2 OS=*Arabidopsis thaliana* GN=TT2 PE=1 SV=1 |
|  | MD15G1222600 | Peroxiredoxin Q, chloroplastic OS=*Populus jackii* GN=PRXQ PE=1 SV=1 |
|  | MD15G1223300 | -- |
|  | MD15G1228900 | Protein PIN-LIKES 5 OS=*Arabidopsis thaliana* GN=PILS5 PE=2 SV=1 |
|  | MD15G1234500 | Pentatricopeptide repeat-containing protein At2g17670 OS=*Arabidopsis thaliana* GN=At2g17670 PE=2 SV=1 |
|  | MD15G1246200 | Naringenin,2-oxoglutarate 3-dioxygenase OS=*Malus domestica* PE=2 SV=1 |
|  | MD15G1260700 | -- |
|  | MD15G1291000 | Probable E3 ubiquitin-protein ligase LUL3 OS=*Arabidopsis* *thaliana* GN=LUL3 PE=1 SV=1 |
|  | MD15G1360000 | Serine hydroxymethyltransferase 7 OS=*Arabidopsis thaliana* GN=SHM7 PE=2 SV=1 |
|  | MD15G1364700 | -- |
|  | MD15G1386300 | Protein ECERIFERUM 2 OS=*Arabidopsis thaliana* GN=CER2 PE=1 SV=1 |
|  | MD15G1408300 | -- |
|  | MD16G1024700 | MLP-like protein 329 OS=*Arabidopsis thaliana* GN=MLP329 PE=2 SV=1 |
|  | MD16G1043200 | Homocysteine S-methyltransferase 1 OS=*Brassica oleracea* var. italica GN=HMT1 PE=1 SV=1 |
|  | MD16G1045500 | -- |
|  | MD16G1048500 | Leucoanthocyanidin reductase OS=*Desmodium uncinatum* GN=LAR PE=1 SV=1 |
|  | MD16G1064700 | -- |
|  | MD16G1088600 | MLP-like protein 423 OS=*Arabidopsis thaliana* GN=MLP423 PE=2 SV=1 |
|  | MD16G1102200 | Inorganic phosphate transporter 2-1, chloroplastic OS=*Arabidopsis thaliana* GN=PHT2-1 PE=1 SV=1 |
|  | MD16G1118100 | Sulfite oxidase OS=*Arabidopsis thaliana* GN=SOX PE=1 SV=1 |
|  | MD16G1125300 | Bidirectional sugar transporter SWEET15 OS=*Vitis vinifera* GN=SWEET15 PE=3 SV=1 |
|  | MD16G1134300 | Diacylglycerol O-acyltransferase 3, cytosolic OS=*Arabidopsis thaliana* GN=DGAT3 PE=1 SV=1 |
|  | MD16G1160600 | Major allergen Pru ar 1 OS=*Prunus armeniaca* PE=1 SV=1 |
|  | MD16G1200000 | Early light-induced protein 1, chloroplastic OS=*Arabidopsis thaliana* GN=ELIP1 PE=1 SV=1 |
|  | MD16G1225200 | Protein PROTON GRADIENT REGULATION 5, chloroplastic OS=*Arabidopsis thaliana* GN=PGR5 PE=1 SV=1 |
|  | MD16G1266400 | Flavonoid 3-O-glucosyltransferase OS=*Medicago truncatula* GN=UGT78G1 PE=1 SV=1 |
|  | MD16G1283100 | PsbP domain-containing protein 3, chloroplastic OS=*Arabidopsis thaliana* GN=PPD3 PE=1 SV=2 |
|  | MD16G1283700 | 2-Cys peroxiredoxin BAS1, chloroplastic OS=*Arabidopsis thaliana* GN=BAS1 PE=1 SV=2 |
|  | MD17G1034000 | Carbonic anhydrase, chloroplastic OS=*Pisum sativum* PE=1 SV=1 |
|  | MD17G1058100 | UDP-glycosyltransferase 83A1 OS=*Arabidopsis thaliana* GN=UGT83A1 PE=2 SV=1 |
|  | MD17G1058200 | UDP-glycosyltransferase 83A1 OS=*Arabidopsis thaliana* GN=UGT83A1 PE=2 SV=1 |
|  | MD17G1090600 | BTB/POZ domain-containing protein At3g22104 OS=*Arabidopsis thaliana* GN=At3g22104 PE=2 SV=1 |
|  | MD17G1155800 | ATPase 10, plasma membrane-type OS=*Arabidopsis thaliana* GN=AHA10 PE=2 SV=2 |
|  | MD17G1214900 | -- |
|  | MD17G1238200 | O-acyltransferase WSD1 OS=*Arabidopsis thaliana* GN=WSD1 PE=2 SV=1 |
|  | MD17G1261000 | Transcription factor MYB113 OS=*Arabidopsis thaliana* GN=MYB113 PE=1 SV=1 |
|  | MD17G1261100 | Transcription factor MYB113 OS=*Arabidopsis thaliana* GN=MYB113 PE=1 SV=1 |
|  | MD17G1272100 | Glutathione S-transferase F12 OS=*Arabidopsis thaliana* GN=GSTF12 PE=1 SV=1 |
|  | Malus_domestica_newGene_7326 | -- |
